# Association between short-term exposure to air pollution and ischemic stroke onset: a time-stratified case-crossover analysis using a distributed lag nonlinear model in Shenzhen, China

**DOI:** 10.1186/s12940-019-0557-4

**Published:** 2020-01-02

**Authors:** Zhinghui Wang, Ji Peng, Peiyi Liu, Yanran Duan, Suli Huang, Ying Wen, Yi Liao, Hongyan Li, Siyu Yan, Jinquan Cheng, Ping Yin

**Affiliations:** 1 Department of Epidemiology and Biostatistics, School of Public Health, Tongji Medical College, Huazhong University of Science and Technology, 13 Hangkong Rd, Wuhan, 430030 Hubei China; 2 Shenzhen Center for Chronic Disease Control, Shenzhen, China; 3 Department of Molecular Epidemiology, Shenzhen Center for Disease Control and Prevention, Shenzhen, China; 4 Department of Occupational and Environment Health, Key Laboratory of Environment and Health, School of Public Health, Tongji Medical College, Huazhong University of Science and Technology, Wuhan, China; 5 Department of Public Health Promotion, Shenzhen Center for Disease Control and Prevention, Shenzhen, China; 6 Shenzhen Center for Disease Control and Prevention, 8 Longyuan Rd, Shenzhen, 518055 Guangdong China

**Keywords:** Ischemic stroke, Air pollution, Case-crossover design, Distributed lag nonlinear model, Quasi-Poisson regression

## Abstract

**Background:**

Stroke, especially ischemic stroke (IS), has been a severe public health problem around the world. However, the association between air pollution and ischemic stroke remains ambiguous.

**Methods:**

A total of 63, 997 IS cases aged 18 years or above in Shenzhen were collected from 2008 to 2014. We used the time-stratified case-crossover design combining with distributed lag nonlinear model (DLNM) to estimate the association between air pollution and IS onset. Furthermore, this study explored the variability across gender and age groups.

**Results:**

The cumulative exposure-response curves were J-shaped for SO_2_, NO_2_ and PM_10_, and V-shaped for O_3_, and crossed over the relative risk (*RR*) of one. The 99th, 50th (median) and 1st percentiles of concentration (μg/m^3^) respectively were 37.86, 10.06, 3.71 for SO_2_, 116.26, 41.29, 18.51 for NO_2_, 145.94, 48.29, 16.14 for PM_10_, and 111.57, 49.82, 16.00 for O_3_. Extreme high-SO_2_, high-NO_2_, high-PM_10_, high-O_3_, and low-O_3_ concentration increased the risk of IS, with the maximum *RR* values and 95% *CI*s: 1.50(1.22, 1.84) (99th vs median) at 0–12 lag days, 1.37(1.13, 1.67) (99th vs median) at 0–10 lag days, 1.26(1.04, 1.53) (99th vs median) at 0–12 lag days, 1.25(1.04, 1.49) (99th vs median) at 0–14 lag days, and 1.29(1.03, 1.61) (1st vs median) at 0–14 lag days, respectively. The statistically significant minimal *RR* value and 95% *CI* was 0.79(0.66,0.94) at 0–10 lag days for extreme low-PM_10_. The elderly aged over 65 years were susceptible to extreme pollution conditions. Difference from the vulnerability of males to extreme high-SO_2_, high-NO_2_ and low-O_3_, females were vulnerable to extreme high-PM_10_ and high-O_3_. Comparing with the elderly, adults aged 18–64 year were immune to extreme low-NO_2_ and low-PM_10_. However, no association between CO and IS onset was found.

**Conclusions:**

SO_2_, NO_2_, PM_10_ and O_3_ exerted non-linear and delayed influence on IS, and such influence varied with gender and age. These findings may have significant public health implications for the prevention of IS.

## Introduction

Stroke has been the second major cause of death and DALYs globally [1, 2]. There were about 10.3 million new strokes and 6.5 million deaths from stroke worldwide in 2013 [3]. In recent years, stroke has been the leading cause of death and DALYs in China [1]. A nationwide population-based survey of 480,687 Chinese adults reported that the age-standardized prevalence, incidence and mortality rates of stroke were 1114.8/100000 people, 246.8/100000 and 114.7/100000 person-years, respectively [4]. IS, which comprises 80% of stroke cases, has had a significant impact on healthcare expenditures and the Chinese economy [5]. Therefore, identification of modifiable risk factors for ischemic stroke would better informed public health prevention work. Exposure to air pollution is the largest environmental health risk and a growing global health problem estimated to contribute to as many as 3.1 million all-cause deaths per year [6–8]. Nevertheless, the impact of air pollution on morbidity from IS might be important and is less certain. Some studies have investigated the associations between short-time exposure to air pollution and stroke onset but no consensus have been reached [9, 10]. The heterogeneity in results may be partially explained by the difference across studies in regions and populations studied, levels, and constituents of air pollution, and sample size or others [11–13]. Besides, most of the studies on the association between air pollution and stroke in China only focused on metropolitan area or heavily polluted regions [14–17]. whereas few studies have been conducted in cities with relatively low levels of air pollution.

Shenzhen, a southern city of the Pearl River Delta region with a sub-tropical maritime climate, is less polluted than most cities in China. While, it is one of the three largest financial centers in China and one of the first-tier cities all over the world. Furthermore, Shenzhen has ranked first in air quality among China’s top 20 cities in GDP in recent years, achieving the development of both air quality and economy. Therefore, we conducted a study on the association between short-time exposure to air pollution and stroke in Shenzhen, an economically advanced and less polluted city of China.

## Methods

### Study population

The data on IS was obtained from 62 major municipal and district general hospitals in Shenzhen, including the National Ministry of Health’s Stroke Prevention and Treatment Base Hospital. The information included basic demographics, date of diagnosis, type of report, and the corresponding *International Classification of Diseases, 10th Revision* (ICD-10) codes and so on. The daily IS stroke count between 1 January 2008 and 31 December 2014 was identified using ICD-10 codes I63. In order to minimize the influence of coding inaccuracy, we used the corresponding diagnosis to check the identified cases. We then reserved the new cases, and individuals aged below 18 years were excluded from this study because of the low incidence.

### Air pollution and meteorological data

Data on air pollution, including levels of sulfur dioxide (SO_2_), nitrogen dioxide (NO_2_), particulate matter less than 10 μm in aerodynamic diameter (PM_10_), carbon monoxide (CO), and ozone (O_3_), were obtained from the Shenzhen Environmental Monitoring Station between 1 January 2008 and 31 December 2014. There were 9 fixed-site air monitoring stations in Shenzhen, located in different districts. Monitoring of air pollution was done in accordance with mandatory quality assurance/quality control (QA/QC) procedures set by the State Environmental Protection Administration of China, ensuring the quality of automatic environmental air monitoring data. We averaged the measurements from all valid monitoring sites. The daily (24 h) mean concentrations of air pollutants were averaged from the available monitoring data across various stations. Data on meteorological factors, including temperature (°C), and relative humidity (%), were obtained from Shenzhen Meteorological Service Center during the period of 1 January 2008 to 31 December 2014.

### Statistical analysis

In this study, we applied the time-stratified case-crossover (ts-CCO) design, regarded as a self-matched case-control study, which compares the exposure in the case period when events occurred with exposures in nearby referent periods, to examine the differences in exposure which may contribute to the differences in the daily count of cases [18]. Therefore, a time-stratified case-crossover design was adopted to regulate potential confounders (e.g., age, gender, etc) using self-control and exclude long-term impact of air pollutants (e.g., secular trend, seasonality, etc.) by stratification of time. We used the calendar month as the time stratum, to control the effects of long-term trend, seasonality, and day of the week [17]. We conducted a quasi-Poisson regression, controlling over-dispersion problem, combined with distributed lag non-linear model (DLNM) to estimate the non-linear and delayed influence of air pollution on IS onset. DLNM is based on the definition of “cross-basis”, a bi-dimensional space of functions to reflect the non-linear exposure-responses and lag structure of the association [19, 20]. In this study, consequently, the combination of DLNM with the ts-CCO design was employed, which allows estimating the short-term, non-linear and delayed effect of air pollutant using cross-basis functions for depicting the relationship between air pollutant and IS onset along the dimensions of exposure and lag simultaneously based on removing control confounders and long-term trend by ts-CCO design.

A quasi-Poisson regression model combined with time-stratified case-crossover design and DLNM was built as follows:

$$ {Y}_t\sim Poisson\left({\mu}_t\right) $$

$$ Log\left({\mu}_t/{Population}_{ye}\right)=\alpha +\sum \limits_{i=1}^m cb\left({Pollutant}_{i,t},{df}_{2i-1},{maxlag}_i,{df}_{2i}\right)+ cb\left({Temp}_t,{df}_{2m+1},{maxlag}_{m+1},{df}_{2m+2}\right)+ cb\left({RH}_t,{df}_{2m+3},{maxlag}_{m+2},{df}_{2m+4}\right)+\gamma {Holiday}_t+\lambda\ Stratum $$

where *t* is the day of observation; *Y*_*t*_ is the count of IS cases on *t*; *μ*_*t*_ is the expectation of *Y*_*t*_; *Population*_*ye*_ is the year-end population size; *α* is an intercept; *Pollutant*_*i*, *t*_, *Temp*_*t*_, and *RH*_*t*_ are the *i*th pollutant concentration, temperature and relative humidity on *t*, respectively; *cb*() represents the cross-basis function with three pre-specified parameters of maximal lag *maxlag*_*i*_, degree of freedom for lag-response natural spline *df*_2*i* − 1_, and degree of freedom for exposure-response natural spline *df*_2*i*_ for pollutant, temperature or relative humidity; *Holiday* is used to control the effect of public holidays; *Stratum* is the time stratum in the time-stratified case-crossover design. We defined natural cubic spline function with 3 df for air pollution and meteorological factors to mimic the exposure-response pattern of air pollution-IS onset associations, as well as lag spaces with 3 df to estimate the lag effects. To capture the complete lag-response curve, the maximal lag of air pollutants was set to 14 days; for the sake of simplification and without loss of generalization, meanwhile, this maximal lag was assigned to the length of the case and control periods. In addition, a 3-day duration was specified to be the maximal lag of meteorological factors. The df and maximum lag days for air pollution determination referred to the Akaike information criterion for quasi-Poisson (Q-AIC), which could produce the relatively superior model.

We initially conducted single-pollutant model to evaluate the association between air pollution and IS onset, and then the significant air pollutants were included in multi-pollutant model. Spearman’s correlation tests were used to estimate the associations between air pollution and meteorological factors, and pollutants with correlation coefficient *r* > 0.60 were not included in multi-pollutant model simultaneously to address the collinearity between air pollutants. In order to identify the high-risk or low-risk air pollution condition, the influence of extreme air pollution was evaluated and presented as relative risk (*RR*) by comparing the 99th above or 1st below percentiles of air pollution to the median values. We calculated the single day lag influence and the cumulative lag influence (lag0–1, lag0–6, lag0–8, lag0–10, lag0–12, lag0–13, and lag0–14) to effectively depict the characteristics of the association between air pollution and IS onset. In addition, we conducted stratified analysis to investigate the impact of air pollution on subgroups according to gender (male and female) and age groups (adult: 18–64 years; the elderly: ≥ 65 years).

All analyses were conducted using R version 3.5.1 with the *dlnm* package for fitting the DLNM, the *gnm* package for conditional quasi-Poisson regression.

Sensitivity analyses were performed to test the robustness of the selected model, which were as following: df [2–6] for air pollution, and df [2–6] for lag space were changed; the maximum lag days (12–21 days) for air pollution were extended.

## Results

The characteristics of study population are presented in Table 1. There were 63,997 IS cases averagely aged 63.41 years met the inclusion criteria for the study between 2008 and 2014, of which 59.49% were males and 48.44% were the elderly.

**Table 1:** Characteristics of the study population in Shenzhen from 2008 to 2014

Characteristics	Total	2008	2009	2010	2011	2012	2013	2014
Gender (n, %)								
Male	38,070 (59.49)	3777 (57.66)	4259 (58.06)	4716 (59.79)	5032 (59.08)	5932 (59.42)	6656 (60.28)	7698 (60.71)
Female	25,927 (40.51)	2774 (42.34)	3077 (41.94)	3172 (40.21)	3485 (40.92)	4051 (40.58)	4386 (39.72)	4982 (39.29)
Age, mean years ^a^	63.41 ± 14.04	63.74 ± 14.42	63.87 ± 14.34	63.35 ± 14.13	63.11 ± 13.92	63.31 ± 13.84	63.35 ± 13.90	63.38 ± 14.00
Age group (n, %)								
18~	6305 (9.85)	706 (10.78)	778 (10.61)	816 (10.34)	836 (9.82)	949 (9.51)	1046 (9.47)	1174 (9.26)
45~	26,692 (41.71)	2492 (38.04)	2766 (37.70)	3252 (41.23)	3661 (42.98)	4296 (43.03)	4727 (42.81)	5498 (43.36)
≥ 65	31,000 (48.44)	3353 (51.18)	3792 (51.69)	3820 (48.43)	4020 (47.20)	4738 (47.46)	5269 (47.72)	6008 (47.38)
Nation								
Han	63,776 (99.68)	6550 (100.00)	7336 (100.00)	7888 (100.00)	8517 (100.00)	9953 (99.70)	10,980 (99.51)	12,552 (99.07)
Minority	202 (0.32)	0 (0.00)	0 (0.00)	0 (0.00)	0 (0.00)	30 (0.30)	54 (0.49)	118 (0.93)
Missing	19	1	0	0	0	0	8	10
District								
Baoan District	15,345 (24.01)	1818 (27.75)	1923 (26.21)	2342 (29.69)	2314 (27.17)	2683 (26.88)	2214 (20.1)	2051 (16.24)
Longgang District	13,917 (21.77)	1392 (21.25)	1554 (21.18)	1608 (20.39)	1860 (21.84)	2223 (22.27)	2522 (22.9)	2758 (21.83)
Futian District	9051 (14.16)	1019 (15.55)	1079 (14.71)	1070 (13.56)	1206 (14.16)	1384 (13.86)	1521 (13.81)	1772 (14.03)
Luohu District	8306 (12.99)	906 (13.83)	930 (12.68)	988 (12.53)	987 (11.59)	1325 (13.27)	1510 (13.71)	1660 (13.14)
Nanshan District	7069 (11.06)	754 (11.51)	961 (13.1)	867 (10.99)	944 (11.08)	1163 (11.65)	1125 (10.21)	1255 (9.93)
Guangming New District	2273 (3.56)	54 (0.82)	185 (2.52)	333 (4.22)	358 (4.2)	385 (3.86)	405 (3.68)	553 (4.38)
Longhua New District	2324 (3.64)	0 (0.00)	0 (0.00)	0 (0.00)	0 (0.00)	0 (0.00)	761 (6.91)	1563 (12.37)
Pingshan New District	1691 (2.65)	129 (1.97)	142 (1.94)	224 (2.84)	254 (2.98)	262 (2.62)	331 (3.00)	349 (2.76)
Yantian District	1039 (1.63)	70 (1.07)	95 (1.29)	143 (1.81)	147 (1.73)	161 (1.61)	203 (1.84)	220 (1.74)
Dapeng New District	154 (0.24)	0 (0.00)	0 (0.00)	0 (0.00)	0 (0.00)	0 (0.00)	46 (0.42)	108 (0.85)
Other	2754 (4.31)	409 (6.24)	467 (6.37)	313 (3.97)	447 (5.25)	397 (3.98)	377 (3.42)	344 (2.72)
Missing	74	0	0	0	0	0	27	47
Education								
Primary school	17,065 (26.67)	1796 (27.42)	2024 (27.59)	2222 (28.17)	2391 (28.07)	2630 (26.34)	2825 (25.59)	3177 (25.06)
Middle school	25,925 (40.51)	2601 (39.71)	3019 (41.15)	3346 (42.42)	3399 (39.91)	3952 (39.59)	4178 (37.84)	5430 (42.83)
High school	11,672 (18.24)	727 (11.1)	1022 (13.93)	1164 (14.76)	1655 (19.43)	1996 (19.99)	2637 (23.88)	2471 (19.49)
Bachelor degree or above	4846 (7.57)	623 (9.51)	561 (7.65)	560 (7.1)	584 (6.86)	717 (7.18)	874 (7.92)	927 (7.31)
Illiteracy	4486 (7.01)	803 (12.26)	710 (9.68)	596 (7.56)	488 (5.73)	688 (6.89)	527 (4.77)	674 (5.32)
Missing	3	1	0	0	0	0	1	1

^a^: mean ± standard deviation

**Fig. 1:**
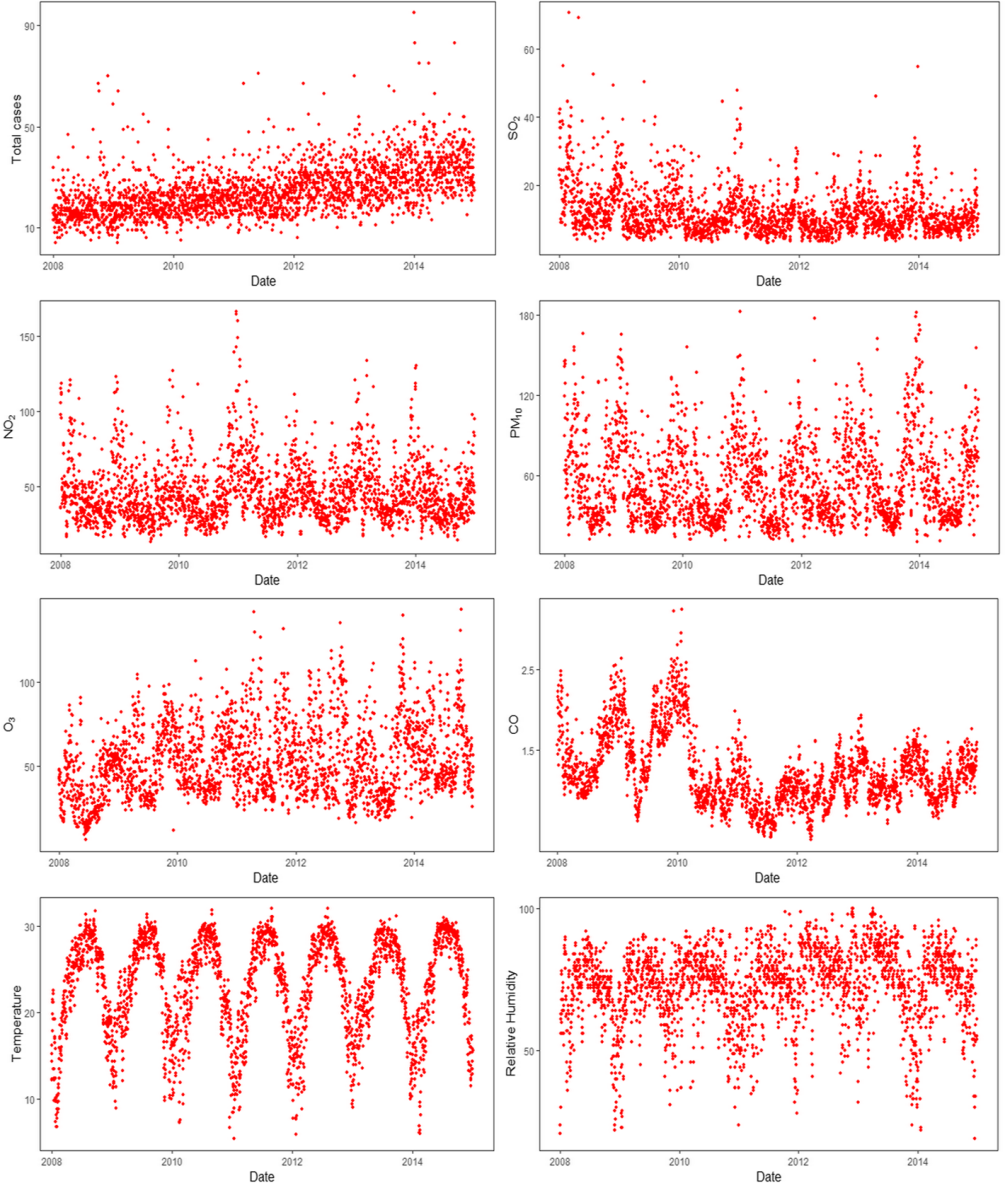
Time-series results regarding the association of ischemic stroke onset with air pollution indicators and meteorological factors in Shenzhen from 2008 to 2014

The summary statistics for daily IS cases, air pollution and meteorological factors in Shenzhen are shown in Table 2. On average, 25 (range: 4–95) IS cases were identified each day during the study period. Of these, there were 15 (range: 1–64) male cases and 10 (range: 1–42) cases, 12 (range: 1–54) elderly cases and 13 (range: 0–42) adult cases respectively. The daily average air pollution levels were 11.97 μg/m^3^ (range: 2.95–70.63 μg/m^3^) for SO_2_, 45.89 μg/m^3^ (range: 13.13–166.14 μg/m^3^) for NO_2_, 55.91 μg/m^3^ (range: 10.86–182.23 μg/m^3^) for PM_10_, 1.22 μg/m^3^ (range: 0.37–3.25 μg/m^3^) for CO, and 53.49 μg/m^3^ (range: 6.02–31.04 μg/m^3^) for O_3_. The means of meteorological factors were 23.03 °C (range: 5.40–32.00 °C) for temperature and 72.83% (range: 19.00–100.00%) for relative humidity. The time-series analysis of IS cases and air pollution and meteorological factors are shown in Fig. 1.

**Table 2:** Descriptions of the day ischemic stroke, air pollution and meteorological factors in Shenzhen from 2008 to 2014

Variables	Mean ± SD	Min	P1	P5	P10	P25	P50	P75	P90	P95	P99	Max	IQR
Total cases	25.03 ± 9.88	4.00	8.00	12.00	14.00	18.00	23.00	31.00	38.00	43.00	53.00	95.00	13.00
Male cases	14.89 ± 6.55	1.00	4.00	6.00	8.00	10.00	14.00	18.00	24.00	27.00	35.00	64.00	8.00
Female cases	10.14 ± 4.63	1.00	2.00	4.00	5.00	7.00	9.00	13.00	16.00	18.00	24.00	42.00	6.00
Elderly cases	12.12 ± 5.51	1.00	3.00	5.00	6.00	8.00	11.00	15.00	19.00	22.00	29.00	54.00	7.00
Adult cases	12.90 ± 5.69	0.00	3.00	5.00	6.00	9.00	12.00	16.00	20.00	23.00	29.00	42.00	7.00
SO_2_(μg/m^3^)	11.97 ± 6.95	2.95	3.71	4.74	5.61	7.35	10.06	14.44	20.35	25.50	37.86	70.63	7.09
NO_2_(μg/m^3^)	45.89 ± 19.96	13.13	18.51	23.17	25.93	32.30	41.29	54.14	72.11	85.04	116.26	166.14	21.84
PM_10_(μg/m^3^)	55.91 ± 30.26	10.86	16.14	21.04	23.96	31.44	48.29	74.66	97.67	114.17	145.94	182.23	43.21
CO (μg/m^3^)	1.22 ± 0.44	0.37	0.50	0.66	0.75	0.91	0.14	1.43	1.88	2.10	2.46	3.25	0.52
O_3_(μg/m^3^)	53.49 ± 21.85	6.02	16.00	23.80	28.89	36.73	49.82	67.78	83.71	93.99	111.57	143.33	31.04
Temperature(°C)	23.03 ± 5.66	5.40	8.80	12.20	14.60	19.00	24.50	27.80	29.30	29.80	30.60	32.00	8.80
Relative humidity(%)	72.83 ± 13.35	19.00	32.00	47.00	54.00	66.00	75.00	82.00	88.00	91.00	97.00	100.00	16.00

Note: *SO*_*2*_ sulfur dioxide; *NO*_*2*_ nitrogen dioxide; *PM*_*10*_ particulate matter less than 10 μm in aerodynamic diameter; *CO* carbon monoxide; *O*_*3*_ ozone; *SD* standard deviation; *Px* percentile of the data; *IQR* inter-quartile range

The correlations between air pollution and meteorological factors are presented in Table 3. The daily concentrations of SO_2_, NO_2_, and PM_10_ were highly and positively correlated with each other (correlation coefficient *r* > 0.6, *P* < 0.0001).

**Table 3:** Spearman correlation coefficients among air pollution and meteorological factors in Shenzhen from 2008 to 2014

Variables	SO_2_	NO_2_	PM_10_	O_3_	CO	Temperature	Relative humidity
SO_2_	1.0000	0.6344	0.7017	0.1404	0.4063	−0.2481	−0.4962
NO_2_	0.6344	1.0000	0.7008	0.0685	0.2926	−0.3123	−0.2382
PM_10_	0.7017	0.7008	1.0000	0.4623	0.3485	−0.3225	− 0.5461
O_3_	0.1404	0.0685	0.4623	1.0000	−0.0101	− 0.0380	− 0.4165
CO	0.4063	0.2926	0.3485	−0.0101	1.0000	−0.3223	− 0.2073
Temperature	−0.2481	− 0.3123	− 0.3225	−0.0380	− 0.3223	1.0000	0.3078
Relative humidity	−0.4962	−0.2382	− 0.5461	−0.4165	− 0.2073	0.3078	1.0000

Note: *SO*_*2*_ sulfur dioxide; *NO*_*2*_ nitrogen dioxide; *PM*_*10*_ particulate matter less than 10 μm in aerodynamic diameter; *CO* carbon monoxide; *O*_*3*_ ozone

The results of the initial single-pollutant models indicated that SO_2_, NO_2_, PM_10_ and O_3_ were associated with IS onset. However, there was no statistical association between CO and IS onset. The single-day and cumulative relative risks of each pollutant in the single-pollutant models are shown in Additional file 1: Figure S1 and Figure S2.

Considering their greater Q-AIC values, the three-pollutant models for the full set combinations of SO_2_, NO_2_, PM_10_ and O_3_ were cleaned out from this study. In view of Q-AIC values of two-pollutant models, and the correlations among SO_2_, NO_2_, and PM_10_, they were included in two-pollutant model with O_3_, separately. Different air pollution variables exerted varied extremely influence on IS cases, and this difference were also observed among subgroups of the population.

**Fig. 2:**
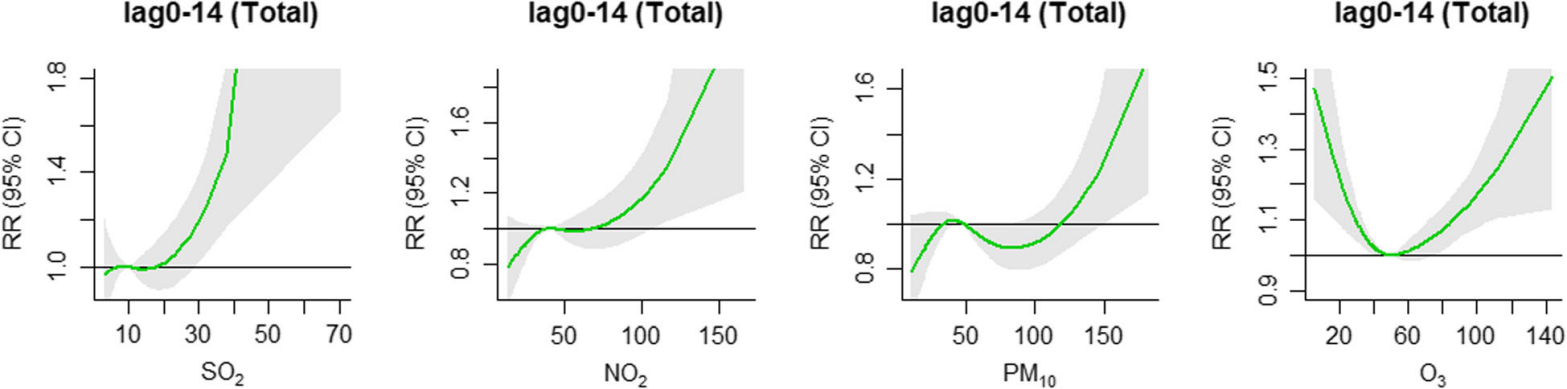
Summary of cumulative exposure-response curves on ischemic stroke for air pollution factors (SO_2_, NO_2_, PM_10_ and O_3_) for total cases at lag0–14 using two-pollutant model in Shenzhen, 2008–2014

The cumulative exposure-response curves for total IS cases at lag0–14 are shown in Fig. 2. The cumulative exposure-response curves were J-shaped for SO_2_, NO_2_ and PM_10._ It can be seen intuitively that only when concentration of SO_2_, NO_2_ and PM_10_ reached a certain limit the influence occurred. In addition, the cumulative lag influence of SO_2_, NO_2_ and PM_10_ were enhanced with the increase of concentration. The cumulative exposure-response curve of O_3_ was V-shaped, which meant that both high and low concentration of O_3_ may increase the risk of IS onset.

**Fig. 3:**
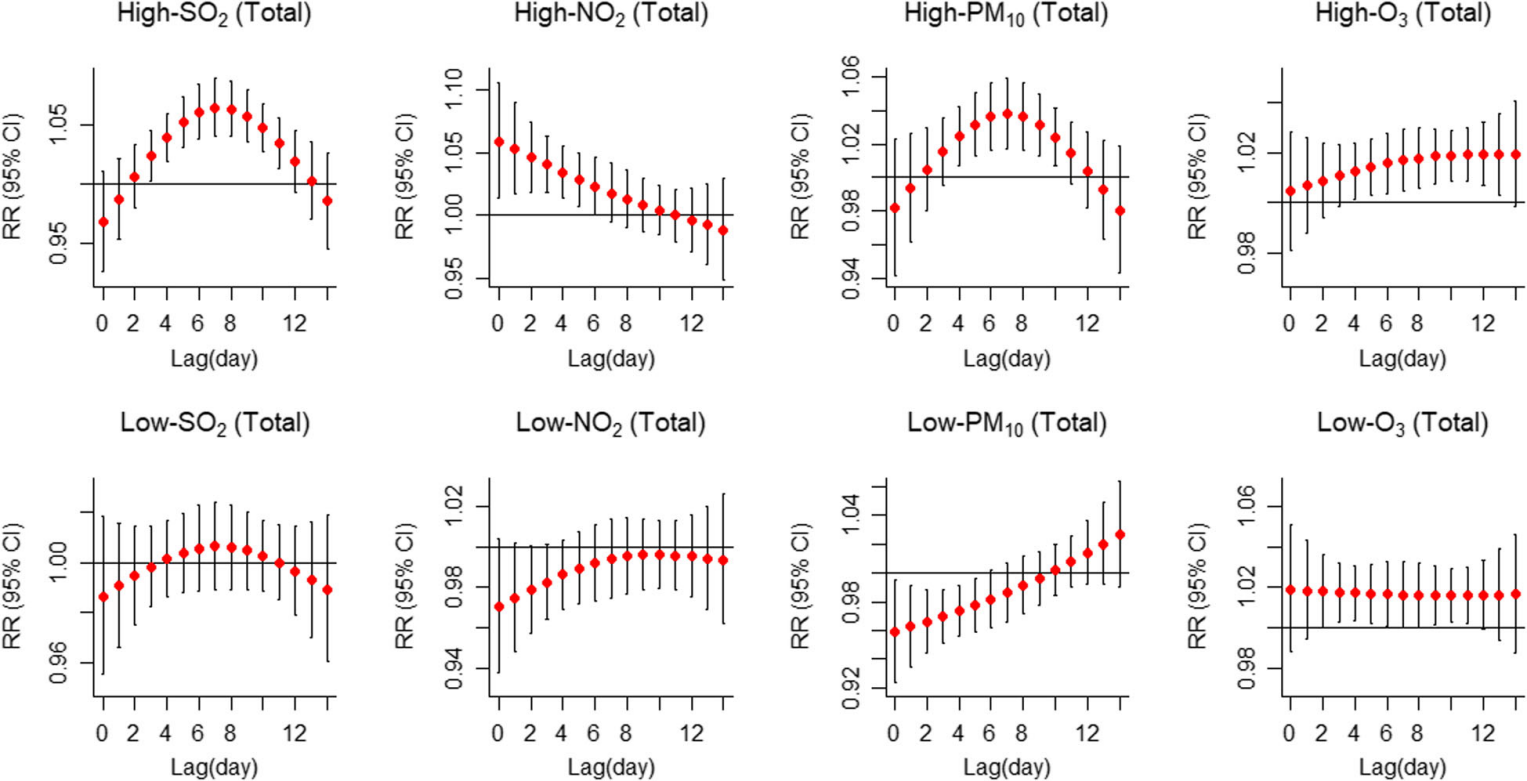
Summary of single day lag-response curves on ischemic stroke for air pollution factors (SO_2_, NO_2_, PM_10_ and O_3_) for total cases at different lags using two-pollutant model in Shenzhen, 2008–2014. The extreme-high influence was estimated by the *RR* of ischemic stroke by comparing the 99th percentile of daily air pollution value to the median value, whereas the extreme-low influence was estimated by comparing the 1st percentile of daily air pollution value to the median value

The single day lag-response curves for total IS cases are shown in Fig. 3. The curve of extreme high-SO_2_ was inverted V-shaped under different lag days, and *RR* value peaked on the eighth lag day and then decreased. No significant association between extreme low-SO_2_ and IS onset was observed at different lag days. The curve of the extreme high-NO_2_ was almost straight line, and the largest *RR* value was observed on the current day and lasted for 6 days. The influence of extreme high-PM_10_ also presented an inverted V-shape curve through the lag days, and the *RR* value reached maximum at lag7 and then decreased. The extreme low-PM_10_ showed a certain protective influence at lag0 to lag6. The extreme high-O_3_ presented statistically significant hazardous influence from lag4 to lag13.

Table 4 summarizes the cumulative influence of extreme air pollution on IS at different lag days for total IS cases. The result showed that extremely high-SO_2_ could significantly increase the risk of IS, and the cumulative influence almost increased with lag days extending, and the maximum *RR* value was 1.50 (1.22, 1.84) (99th vs median), appearing at lag0–12. No prominently cumulative influence of extreme low-SO_2_ on the IS onset was observed at different lag days. Extreme high-NO_2_ was significant associated with IS, showing a hazardous influence, and the maximum *RR* value was 1.37 (1.13, 1.67) (99th vs median) at lag0–10. The influence of extreme low -NO_2_ was not observed in total group. Extreme high-PM_10_ can increase the risk of IS onset, and the maximum *RR* value was 1.26(1.04, 1.53) (99th vs median) at lag0–12. Extreme low-PM_10_ showed a protective influence on IS onset, and the minimal *RR* value was 0.79(0.66,0.94) at lag0–10. Both extreme high-O_3_ and low-O_3_ could increase the risk of IS, regardless of whether it was statistically significant or not, and the maximum *RR* values were 1.25(1.04, 1.49) (99th vs median) at lag0–14 and 1.29(1.03, 1.61) (1st vs median) at lag0–14, respectively.

**Table 4:** Extreme influence analysis of different air pollution factors from 2008 to 2014 using two-pollutant models. Relative risk (*RR*) and 95% confidence interval (*CI*) were used to estimate the cumulative influence of air pollution factors in total IS cases

pollutant	extreme-low influence						extreme-high influence					
	lag0–6	lag0–8	lag0–10	lag0–12	lag0–13	lag0–14	lag0–6	lag0–8	lag0–10	lag0–12	lag0–13	lag0–14
SO2	0.98 (0.87,1.10)	0.99 (0.87,1.13)	1.00 (0.86,1.16)	0.99 (0.85,1.17)	0.99 (0.84,1.17)	0.98 (0.82,1.17)	1.14 (0.98,1.32)	1.29 (1.09,1.52)	1.42 (1.18,1.71)	1.50 (1.22,1.84)	1.50 (1.21,1.86)	1.48 (1.17,1.87)
NO2	0.88 (0.77,1.00)	0.87 (0.75,1.01)	0.86 (0.73,1.03)	0.86 (0.71,1.04)	0.85 (0.70,1.04)	0.85 (0.68,1.05)	1.32 (1.13,1.54)	1.36 (1.14,1.62)	1.37 (1.13,1.67)	1.37 (1.10,1.70)	1.36 (1.08,1.71)	1.34 (1.05,1.72)
PM10	0.81 (0.71,0.92)	0.79 (0.68,0.92)	0.79 (0.66,0.94)	0.81 (0.67,0.97)	0.82 (0.67,1.00)	0.84 (0.68,1.04)	1.09 (0.95,1.26)	1.17 (1.00,1.37)	1.24 (1.04,1.47)	1.26 (1.04,1.53)	1.25 (1.02,1.54)	1.23 (0.98,1.53)
O3	1.13 (0.97,1.32)	1.17 (0.99,1.38)	1.21 (1.00,1.45)	1.24 (1.02,1.52)	1.27 (1.03,1.56)	1.29 (1.03,1.61)	1.07 (0.94,1.22)	1.11 (0.96,1.28)	1.15 (0.99,1.35)	1.20 (1.01,1.42)	1.22 (1.03,1.45)	1.25 (1.04,1.49)

Note: Estimates were generated using a quasi-Poisson regression model combined with time-stratified case-crossover design and distributed lag non-linear model (DLNM), adjusting for meteorological factors, holiday, and time stratum. The extreme-high influence was estimated by the *RR* of ischemic stroke by comparing the 99th percentile of daily air pollution value to the median value, whereas the extreme-low influence was estimated by comparing the 1st percentile of daily air pollution value to the median value

**Fig. 4:**
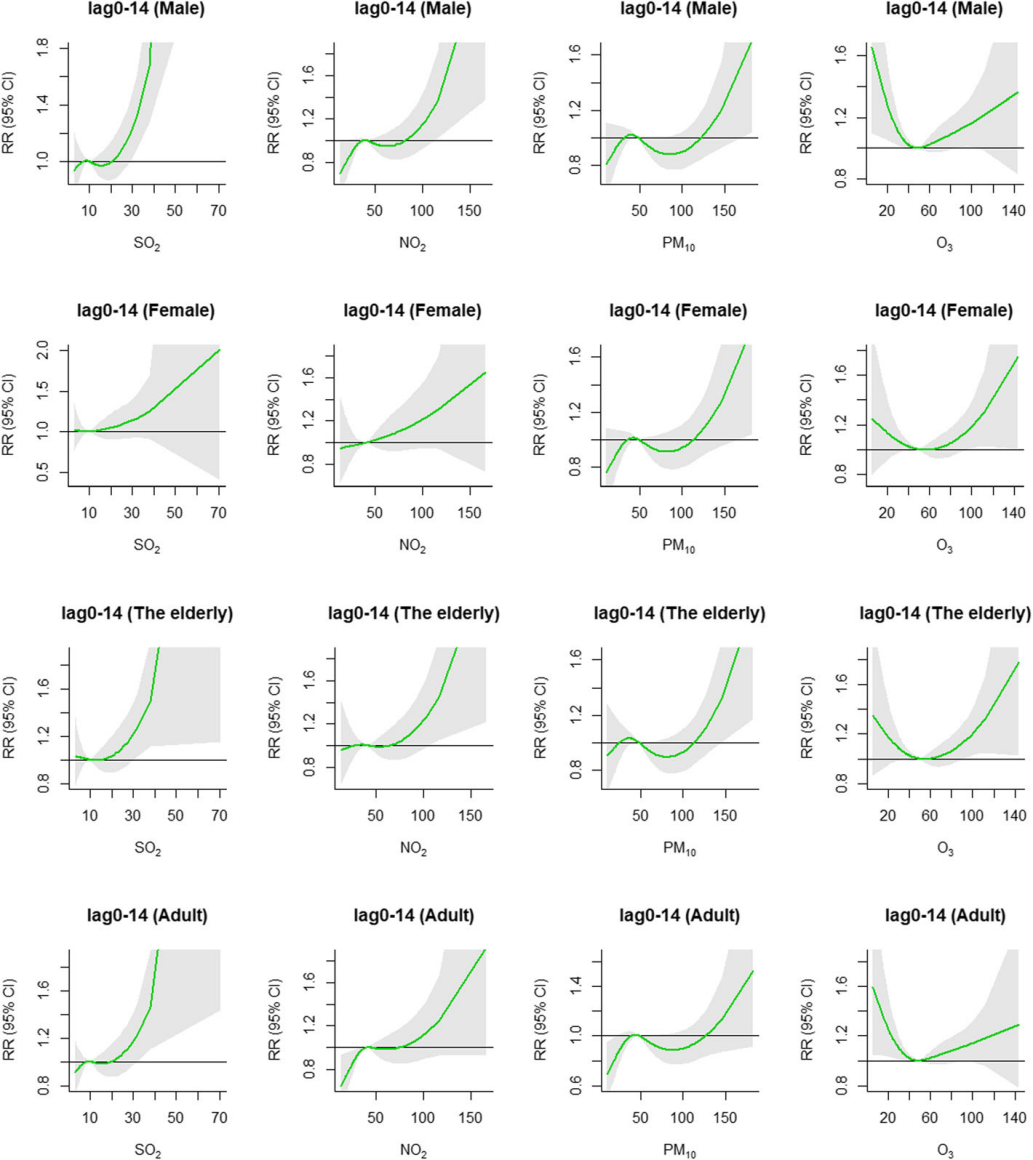
Summary of cumulative exposure-response curves on ischemic stroke for air pollution factors (SO_2_, NO_2_, PM_10_ and O_3_) for subgroups at lag0–14 using two-pollutant model in Shenzhen, 2008–2014. Male and female were subgroups according to gender. The elderly and adult were subgroups according to age (adult: 18–64 years; the elderly: ≥ 65 years)

The cumulative exposure-response curves for subgroups shown in Fig. 4 were similar with total IS cases. It indicated that SO_2_ showed stronger influence on males and adults. The influence of NO_2_ and PM_10_ was a little stronger in males and the elderly than in females and adults. Yet, the impact of O_3_ shows almost no difference among males and adults, females and the elderly respectively.

The single day lag-response curves of subgroups presented in Additional file 1: Figure S3 resembled these of total IS cases.

The cumulative influence of extreme air pollution on IS at different lag days for subgroups are shown in Table 5. Males were more susceptible to extreme high-SO_2_ than females, and the maximum *RR* value is 40% higher. Extreme high-SO_2_ had a stronger influence on the elderly and adults. However, no prominently cumulative influence of extreme low-SO_2_ on subgroups was observed at different lag days. The influence of extreme high-NO_2_ on males was slightly stronger than females, and the *RR* value was 0.19 higher. Extreme high-NO_2_ showed a strong influence on the elderly, but insignificantly on adults. Extreme low-NO_2_ presented protective influence on males and adults. Females and the elderly were more sensitive for extreme high-PM_10_. Extreme low-PM_10_ showed protective influence, especially on females and adults. Extreme high-O_3_ showed significant risk influence in females and the elderly. Besides, extreme low-O_3_ showed significant risk influence on males and adults.

**Table 5:** Extreme influence analysis of different air pollution factors from 2008 to 2014 using two-pollutant models. Relative risk (*RR*) and 95% confidence interval (*CI*) were used to estimate the cumulative influence of air pollution factors in subgroups

pollutant	population	extreme-low influence						extreme-high influence					
		lag0–6	lag0–8	lag0–10	lag0–12	lag0–13	lag0–14	lag0–6	lag0–8	lag0–10	lag0–12	lag0–13	lag0–14
SO2	Male	0.99 (0.87,1.13)	1.01 (0.87,1.17)	1.01 (0.86,1.19)	0.99 (0.83,1.19)	0.98 (0.81,1.18)	0.95 (0.78,1.17)	1.27 (1.07,1.52)	1.47 (1.22,1.79)	1.65 (1.33,2.04)	1.73 (1.37,2.18)	1.72 (1.34,2.20)	1.67 (1.28,2.19)
	Female	0.97 (0.83,1.12)	0.97 (0.82,1.15)	0.98 (0.81,1.19)	1.00 (0.81,1.23)	1.01 (0.81,1.25)	1.02 (0.80,1.28)	0.97 (0.80,1.17)	1.06 (0.86,1.32)	1.16 (0.91,1.47)	1.22 (0.94,1.59)	1.24 (0.94,1.64)	1.24 (0.92,1.68)
	The elderly	1.02 (0.88,1.18)	1.04 (0.88,1.23)	1.05 (0.87,1.27)	1.05 (0.86,1.29)	1.04 (0.84,1.29)	1.03 (0.82,1.29)	1.14 (0.94,1.37)	1.31 (1.07,1.62)	1.47 (1.17,1.85)	1.55 (1.20,1.99)	1.54 (1.18,2.01)	1.49 (1.12,2.00)
	Adult	0.95 (0.83,1.08)	0.95 (0.82,1.10)	0.95 (0.80,1.12)	0.95 (0.79,1.13)	0.94 (0.78,1.14)	0.93 (0.76,1.15)	1.13 (0.95,1.35)	1.26 (1.03,1.53)	1.37 (1.10,1.71)	1.45 (1.14,1.85)	1.47 (1.13,1.89)	1.46 (1.11,1.93)
NO2	Male	0.84 (0.73,0.98)	0.83 (0.69,0.98)	0.81 (0.67,0.99)	0.80 (0.64,0.99)	0.79 (0.63,0.99)	0.78 (0.61,1.00)	1.39 (1.16,1.66)	1.43 (1.17,1.75)	1.45 (1.15,1.81)	1.43 (1.11,1.83)	1.40 (1.08,1.83)	1.37 (1.03,1.83)
	Female	0.94 (0.79,1.12)	0.95 (0.78,1.15)	0.95 (0.76,1.19)	0.96 (0.75,1.22)	0.96 (0.74,1.24)	0.96 (0.72,1.26)	1.23 (1.00,1.50)	1.26 (1.01,1.58)	1.28 (0.99,1.65)	1.29 (0.98,1.71)	1.30 (0.96,1.75)	1.30 (0.94,1.79)
	The elderly	0.95 (0.80,1.12)	0.95 (0.78,1.15)	0.95 (0.77,1.18)	0.96 (0.76,1.22)	0.97 (0.75,1.24)	0.97 (0.74,1.28)	1.49 (1.23,1.81)	1.54 (1.23,1.91)	1.54 (1.20,1.96)	1.50 (1.14,1.98)	1.48 (1.11,1.97)	1.44 (1.06,1.98)
	Adult	0.82 (0.70,0.95)	0.80 (0.67,0.96)	0.79 (0.65,0.96)	0.77 (0.62,0.96)	0.75 (0.60,0.95)	0.74 (0.58,0.95)	1.16 (0.97,1.40)	1.20 (0.98,1.48)	1.23 (0.98,1.55)	1.24 (0.97,1.60)	1.25 (0.95,1.63)	1.24 (0.93,1.66)
PM10	Male	0.81 (0.70,0.95)	0.79 (0.66,0.94)	0.78 (0.64,0.95)	0.80 (0.65,1.00)	0.83 (0.66,1.04)	0.86 (0.67,1.10)	1.12 (0.95,1.32)	1.19 (1.00,1.43)	1.24 (1.02,1.51)	1.25 (1.01,1.55)	1.23 (0.97,1.55)	1.20 (0.93,1.55)
	Female	0.80 (0.67,0.95)	0.80 (0.65,0.97)	0.80 (0.64,1.00)	0.81 (0.64,1.04)	0.82 (0.63,1.05)	0.82 (0.62,1.08)	1.04 (0.87,1.25)	1.14 (0.93,1.40)	1.23 (0.99,1.54)	1.28 (1.01,1.64)	1.29 (0.99,1.67)	1.27 (0.95,1.69)
	The elderly	0.87 (0.73,1.03)	0.86 (0.71,1.05)	0.87 (0.70,1.08)	0.89 (0.70,1.13)	0.91 (0.71,1.18)	0.94 (0.72,1.23)	1.15 (0.97,1.37)	1.27 (1.04,1.54)	1.36 (1.10,1.68)	1.38 (1.09,1.75)	1.36 (1.06,1.76)	1.32 (1.00,1.74)
	Adult	0.75 (0.64,0.88)	0.73 (0.61,0.87)	0.72 (0.59,0.88)	0.73 (0.59,0.91)	0.74 (0.59,0.94)	0.76 (0.60,0.97)	1.03 (0.87,1.22)	1.08 (0.90,1.30)	1.13 (0.92,1.38)	1.15 (0.92,1.44)	1.14 (0.90,1.46)	1.13 (0.87,1.47)
O3	Male	1.22 (1.03,1.46)	1.27 (1.05,1.55)	1.32 (1.06,1.63)	1.35 (1.07,1.70)	1.37 (1.07,1.74)	1.38 (1.07,1.79)	1.06 (0.91,1.23)	1.09 (0.92,1.29)	1.13 (0.94,1.35)	1.17 (0.97,1.42)	1.19 (0.98,1.45)	1.21 (0.98,1.50)
	Female	1.01 (0.83,1.23)	1.03 (0.83,1.28)	1.06 (0.84,1.35)	1.11 (0.86,1.43)	1.14 (0.87,1.48)	1.17 (0.88,1.55)	1.10 (0.93,1.30)	1.15 (0.95,1.38)	1.20 (0.98,1.46)	1.25 (1.01,1.54)	1.27 (1.02,1.59)	1.30 (1.03,1.64)
	The elderly	1.05 (0.87,1.28)	1.07 (0.86,1.32)	1.09 (0.86,1.38)	1.14 (0.89,1.47)	1.18 (0.91,1.53)	1.22 (0.93,1.62)	1.07 (0.91,1.27)	1.13 (0.94,1.36)	1.19 (0.98,1.46)	1.26 (1.02,1.55)	1.29 (1.03,1.60)	1.32 (1.05,1.66)
	Adult	1.21 (1.02,1.45)	1.28 (1.05,1.55)	1.32 (1.07,1.64)	1.35 (1.07,1.70)	1.35 (1.06,1.72)	1.34 (1.04,1.74)	1.07 (0.93,1.24)	1.09 (0.93,1.29)	1.12 (0.93,1.34)	1.15 (0.95,1.39)	1.16 (0.95,1.42)	1.18 (0.96,1.45)

Note: Estimates were generated using a quasi-Poisson regression model combined with time-stratified case-crossover design and distributed lag non-linear model (DLNM), adjusting for meteorological factors, holiday, and time stratum. Male and female were subgroups according to gender. The extreme-high influence was estimated by the *RR* of ischemic stroke by comparing the 99th percentile of daily air pollution value to the median value, whereas the extreme-low influence was estimated by comparing the 1st percentile of daily air pollution value to the median value. The elderly and adult were subgroups according to age (adult: 18–64 years; the elderly: ≥ 65 years)

To facilitate comparison with previous studies, the *RR*s and 95% *CI*s of air pollution factors on IS onset at lag0, lag1 and lag0–1 are listed in Additional file 1: Table S1. In addition, the cumulative exposure-response curves of air pollutants on IS at lag0–1, lag0–6 and lag0–14 are presented in Additional file 1: Figure S4.

The results of sensitivity analysis, which were conducted by changing the df for air pollution from 2 to 6, the df for the lag space from 2 to 6, and by changing the maximum lag days from 12 to 21 days, were similar to the results obtained above. Results of sensitivity analysis were shown in Additional file 1: Figure S5-S10.

## Discussion

In this study, we found significant association between short-term exposure to SO_2_, NO_2_, PM_10_, and O_3_ and IS onset. The influence of exposing to high-level and low-level air pollution was not concerted for subgroups depending on gender and age.

The present study found that extreme high-SO_2_, high-NO_2_, and high-PM_10_ could increase the risk of IS. A multicity case-crossover study found significantly positive associations between SO_2_ [*RR* and 95% confidence interval (*CI*): 1.016(01.0100, 1.0230) per interquartile range (IQR) increase], NO_2_ [*RR* and 95% *CI*: 1.0260(1.0180, 1.0350) per IQR increase], PM_10_ [*RR* and 95% *CI*: 1.0070(1.000, 1.0140) per IQR increase] and IS [21]. Another multicity study reported that SO_2_ [*RR* and 95% *CI*: 1.0135 (1.0043, 1.0229) per IQR increase], NO_2_ [*RR* and 95% *CI*: 1.0294 (1.0178, 1.0412) per IQR increase] and PM_10_ [*RR* and 95% *CI*: 1.0103 (1.0004, 1.0204) per IQR increase] were significantly associated with IS onset [22]. In addition, meta-analyses also found significantly positive associations between SO_2_, NO_2_, PM_10_ and stroke [9, 10]. Previous studies used different study designs and model specifications, which made us unable to compare the non-linear and lag influence across cities. However, these findings of associations between high concentration and IS onset were similar as a whole. Thus, the associations between the three kinds of air pollutants and IS were unlikely to be mendacious because of potential confounding, defects of the design or statistical analysis, or publication and reporting bias. Conflicting evidences were found on the modification by gender or age in the associations between air pollution and IS [16, 21, 23, 24], and the underlying mechanisms were ambiguous.

Biologic mechanisms for these associations above have not been fully established, and majority of previous studies focused on particulate matter (PM). Several potential mechanisms have been proposed including systemic inflammation [25, 26], thrombosis [27–29], artery calcification [30], and vascular endothelial dysfunction [31], induced by exposure air pollution. The acute systemic inflammatory response with increased plasma fibrinogen, C-reactive protein and white blood cell [25, 32, 33], could be a trigger for inflammation and thrombokinesis. These air-pollution-related pathophysiologic changes may be associated with the occurrence of IS.

We found no association between CO and IS onset, although it was controversial in earlier studies. A study of 9 cities suggested a positive association between CO (*RR* and 95% *CI*: 1.0283 (1.0123, 1.0446) per 0.3 ppm increase) and IS onset in single-pollutant model [22], while the association may not persist when adjusted by other pollutants. Another study conducted in Copenhagen, Denmark found ambient CO was associated with an increased risk of IS in single-pollutant model and the association was attenuated to null after adjusting for PM [34]. In Taiwan, significant positive association was found between CO and IS admission in the single-pollutant model, but it became insignificant when controlled for other pollutants [35]. The lack of multi-pollutant models may distort the association. A time series study in Hong Kong found a protective influence between CO [*RR* and 95% *CI* was 0.980 (0.967, 0.993) per 0.3 ppm increase] and stroke admission [36]. However, the study did not differentiate between ischemic and hemorrhagic stroke, so it may be not sure that whether the association exists when we limited the outcome to IS. Also, Also, there are several other multicity-based studies in China found no association between CO and IS [21, 37], which supported our results.

The cumulative dose-response relationship between O_3_ and IS onset was V-shaped, which meant that higher or lower levels of O_3_ concentrations may both increase the risk of IS. However, evidences from previous studies were incongruous. Several studies reported significantly positive association between O_3_ and IS onset [38–40], or stroke emergency hospital visits [41]. Nevertheless, some other studies found no association between O_3_ and stroke incidence [11, 41–43]. One possible explanation for the inconsistent findings was that O_3_ may increase the risk of IS onset among susceptible or vulnerable populations [44]. Several other studies found insignificant negative association between O_3_ and stroke onset, with estimation of *OR* with 95% *CI* as 0.97(0.87, 1.07) per 10 ppb increase in Nueces County, Texas [42], and 0.98(0.96, 1.01) per 10 ppb increase in South Carolina [43], respectively. A recent study in Changzhou, China reported a protective influence of ambient O_3_ on stroke onset, and the *RR* with 95% *CI* was 0.9966(0.9944, 0.9988) per IQR increase [45]. Major ozonated auto-hemo-therapy has been used in the treatment in ischemic disorders, including acute cerebral infarction [46, 47]. In addition, the neuroprotective dose-response curve for O_3_ after a stroke was shaped as U with effective level range of 80–120 μg/mL in rat models [48]. Therefore, the consistency of this biological evidence with our epidemiological results indicates necessity of conducting more investigations to validate complicate association of O_3_ with IS and then to determine range of low-risk O_3_ concentration.

There are some strengths of the present study. First, the data on IS were derived from the majority of general hospitals with the ability to diagnose and treat stoke in Shenzhen, and the sample size was relatively large. Therefore, the present results may be representative of the authentic associations between air pollution and IS in the study area. Second, we only included the new IS cases, as exclusion of the recurrent cases can better reflect the influence of air pollution on IS onset. Third, as far as we know, it was the first time that a time-stratified case-crossover design combining with DLNM has been used to study the between air pollution and IS onset. The case-crossover design is a reliable method to estimate the short-term health influence of exposure to air pollution [49], and the DLNM can better depict the non-linear and delayed influence of air pollution on IS onset. In addition, we found the V-shaped relationship between O_3_ and IS for the first time, which may important implications for the prevention and treatment of IS. However, several limitations should be addressed. Firstly, the present results were derived from data of only one city. Although Shenzhen is a representative of cities with advanced economy and low levels of air pollution, it should be cautious to extrapolate our results to other areas. Secondly, the date of diagnosis, rather than the time of stroke symptom onset, was used in the analysis, which may result in temporal misalignment between air pollution exposure and IS incidence and the underestimate of exposure effects [50]. However, because stoke is a kind of disease requiring prompt hospitalization and treatment, temporal misalignment is expected to play a minor role in our estimation. As the admission or diagnosis may occur in the days after onset of symptoms, it should be gingerly to explain the lag influence of air pollution on IS onset. Finally, the data on air pollution was evaluated using the arithmetic mean from 9 fixed stations, but not individual exposure, which may cause exposure measurement error, leading to underestimation of the influence of air pollution [51].

Although the increased risk of IS triggered by air pollution is relatively small for each individual, the public health implications are very important because a large number of populations are at risk for IS and exposed to unavoided and modifiable air pollution. Further studies with more accurate time of stroke symptom onset are needed to further validate our findings. Additional studies should be conducted to investigate the specific components of air pollutants that play a role in the association between air pollution and IS onset.

## Conclusions

In conclusion, our study suggested that the short-term exposure to SO_2_, NO_2_, and PM_10_ was significantly associated with increased IS risk, except for CO. The cumulative exposure-response curve between O_3_ and IS onset was V-shaped, which may provide an epidemiological evidence for the neuroprotective function. These findings may have significant public health implications for the prevention of IS. Further studies on this topic are warranted to validate our research.

## Additional file

**Additional file 1.** Supplemental materials.

## Data Availability

The datasets generated and analyzed during the current study are not publicly available due to no permission but are available from the corresponding author on reasonable request.

## Abbreviations

CI: Confidence interval
DLNM: Distributed lag nonlinear model
ICD-10: *International Classification of Diseases, 10th Revision*
IQR: Interquartile range
IS: Ischemic stroke
*RR*: Relative risk
