# Supplementary material for: Association between short-term exposure to air pollution and ischemic stroke onset: a time-stratified case-crossover analysis using a distributed lag nonlinear model in Shenzhen, China
Source: Environ Health. 2020 Jan 2;19:1. doi: 10.1186/s12940-019-0557-4 (PMC6941275; doi:10.1186/s12940-019-0557-4)
Supplement: Supplementary file 1 — Additional file 1. Supplemental materials. [file 12940_2019_557_MOESM1_ESM.pdf]

Supplemental Material

Association between short-term exposure to air pollution and ischemic stroke onset: a time-stratified case-crossover analysis using a distributed lag nonlinear model in Shenzhen, China

Zhinghui Wang, Ji Peng, Peiyi Liu, Yanran Duan, Suli Huang, Ying Wen, Yi Liao, Hongyan Li, Siyu Yan, Jinquan Cheng, Ping Yin

Table of Contents

Table S1 Extreme influence analysis of different air pollution factors from 2008 to 2014 using two-pollutant models. Relative risk (*RR*) and 95% confidence interval (*CI*) were used to estimate the single lag day and cumulative influence of air pollution factors in total cases.

Figure S1 Summary of single day lag-response curves on ischemic stroke for air pollution factors (SO_2, NO_2, PM_{10} and O_3) for all groups at different lags using single-pollutant model in Shenzhen, 2008-2014. The extreme-high influence was estimated by the *RR* of ischemic stroke by comparing the 99th percentile of daily air pollution value to the median value, whereas the extreme-low influence was estimated by comparing the 1st percentile of daily air pollution value to the median value. The elderly and adult were subgroups according to age (adult: 18-64 years; the elderly: ≥ 65 years).

Figure S2 Summary of cumulative exposure-response curves on ischemic stroke for air pollution factors (SO_2, NO_2, PM_{10} and O_3) for all groups at lag0-14 using single-pollutant model in Shenzhen, 2008-2014. Male and female were subgroups according to gender. The elderly and adult were subgroups according to age (adult: 18-64 years; the elderly: ≥ 65 years).

Figure S3 Summary of single day lag-response curves on ischemic stroke for air pollution factors (SO_2, NO_2, PM_{10} and O_3) for subgroups at different lags using two-pollutant model in Shenzhen, 2008-2014. The extreme-high influence was estimated by the *RR* of ischemic stroke by comparing the 99th percentile of daily air pollution value

to the median value, whereas the extreme-low influence was estimated by comparing the 1st percentile of daily air pollution value to the median value. The elderly and adult were subgroups according to age (adult: 18-64 years; the elderly: ≥ 65 years).

Figure S4 Summary of cumulative exposure-response curves on ischemic stroke for air pollution factors (SO_2, NO_2, PM_{10} and O_3) for total cases at lag0-1, lag0-6 and lag0-14 using two-pollutant model in Shenzhen, 2008-2014.

Figure S5 Summary of single day lag-response curves on ischemic stroke for air pollution factors (SO_2, NO_2, PM_{10} and O_3) for total cases at different lags using two-pollutant model by changing the df (2-6) of air pollution in Shenzhen, 2008-2014. The extreme-high influence was estimated by the *RR* of ischemic stroke by comparing the 99th percentile of daily air pollution value to the median value, whereas the extreme-low influence was estimated by comparing the 1st percentile of daily air pollution value to the median value.

Figure S6 Summary of cumulative exposure-response curves on ischemic stroke for air pollution factors (SO_2, NO_2, PM_{10} and O_3) for total cases at lag0-14 using two-pollutant model by changing the df (2-6) of air pollution in Shenzhen, 2008-2014.

Figure S7 Summary of single day lag-response curves on ischemic stroke for air pollution factors (SO_2, NO_2, PM_{10} and O_3) for total cases at different lags using two-pollutant model by changing the df (2-6) of lag space in Shenzhen, 2008-2014. The extreme-high influence was estimated by the *RR* of ischemic stroke by comparing the 99th percentile of daily air pollution value to the median value, whereas the extreme-low influence was estimated by comparing the 1st percentile of daily air pollution value to the median value.

Figure S8 Summary of cumulative exposure-response curves on ischemic stroke for air pollution factors (SO_2, NO_2, PM_{10} and O_3) for total cases at lag0-14 using two-pollutant model by changing the df (2-6) of lag space in Shenzhen, 2008-2014.

Figure S9 Summary of single day lag-response curves on ischemic stroke for air pollution factors (SO_2, NO_2, PM_{10} and O_3) for total cases at different lags using two-pollutant model by changing the maximum lag days (12-21 days) for air pollution in Shenzhen, 2008-2014. The extreme-high influence was estimated by the *RR* of ischemic stroke by comparing the 99th percentile of daily air pollution value to the median value, whereas the extreme-low influence was estimated by comparing the 1st percentile of daily air pollution value to the median value.

Figure S10 Summary of cumulative exposure-response curves on ischemic stroke for air pollution factors (SO_2, NO_2, PM_{10} and O_3) for total cases at lag0-14 using two-pollutant model by changing the maximum lag days (12-21 days) for air pollution in Shenzhen, 2008-2014.

Table S1 Extreme influence analysis of different air pollution factors from 2008 to 2014 using two-pollutant models. Relative risk (*RR*) and 95% confidence interval (*CI*) were used to estimate the single lag day and cumulative influence of air pollution factors in total cases.

pollutant	extreme-low influence			extreme-high influence		
	lag0	lag1	lag0-1	lag0	lag1	lag0-1
SO_2	0.99(0.96,1.02)	0.99(0.97,1.02)	0.98(0.92,1.03)	0.97(0.93,1.01)	0.99(0.95,1.02)	0.95(0.88,1.03)
NO_2	0.97(0.94,1.00)	0.97(0.95,1.00)	0.95(0.89,1.01)	1.06(1.01,1.11)	1.05(1.02,1.09)	1.11(1.03,1.20)
PM_{10}	0.96(0.92,1.00)	0.96(0.93,0.99)	0.92(0.86,0.99)	0.98(0.94,1.02)	0.99(0.96,1.03)	0.97(0.91,1.05)
O_3	1.02(0.99,1.05)	1.02(0.99,1.04)	1.04(0.96,1.13)	1.01(0.98,1.03)	1.01(0.99,1.03)	1.01(0.95,1.08)

Note: Estimates were generated using a quasi-Poisson regression model combined with time-stratified case-crossover design and distributed lag non-linear model (DLNM), adjusting for meteorological factors, holiday, and time stratum. The extreme-high influence was estimated by the *RR* of ischemic stroke by comparing the 99th percentile of daily air pollution value to the median value, whereas the extreme-low influence was estimated by comparing the 1st percentile of daily air pollution value to the median value.

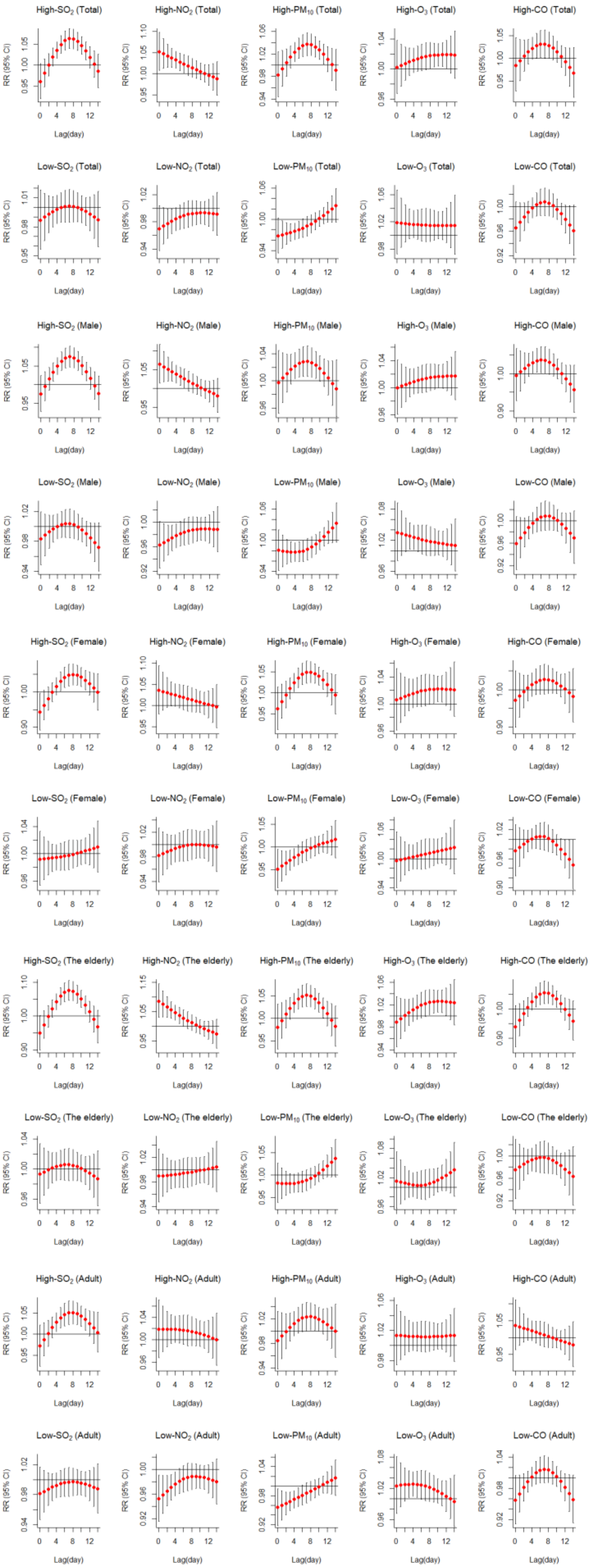
High-SO_2 (Total)
High-NO_2 (Total)
High-PM_{10} (Total)
High-O_3 (Total)
High-CO (Total)
Low-SO_2 (Total)
Low-NO_2 (Total)
Low-PM_{10} (Total)
Low-O_3 (Total)
Low-CO (Total)
High-SO_2 (Male)
High-NO_2 (Male)
High-PM_{10} (Male)
High-O_3 (Male)
High-CO (Male)
Low-SO_2 (Male)
Low-NO_2 (Male)
Low-PM_{10} (Male)
Low-O_3 (Male)
Low-CO (Male)
High-SO_2 (Female)
High-NO_2 (Female)
High-PM_{10} (Female)
High-O_3 (Female)
High-CO (Female)
Low-SO_2 (Female)
Low-NO_2 (Female)
Low-PM_{10} (Female)
Low-O_3 (Female)
Low-CO (Female)
High-SO_2 (The elderly)
High-NO_2 (The elderly)
High-PM_{10} (The elderly)
High-O_3 (The elderly)
High-CO (The elderly)
Low-SO_2 (The elderly)
Low-NO_2 (The elderly)
Low-PM_{10} (The elderly)
Low-O_3 (The elderly)
Low-CO (The elderly)
High-SO_2 (Adult)
High-NO_2 (Adult)
High-PM_{10} (Adult)
High-O_3 (Adult)
High-CO (Adult)
Low-SO_2 (Adult)
Low-NO_2 (Adult)
Low-PM_{10} (Adult)
Low-O_3 (Adult)
Low-CO (Adult)
RR (95% CI)
Lag(day)

Figure S1 Summary of single day lag-response curves on ischemic stroke for air pollution factors (SO_2, NO_2, PM_{10} and O_3) for all groups at different lags using single-pollutant model in Shenzhen, 2008-2014. The extreme-high influence was estimated by the *RR* of ischemic stroke by comparing the 99th percentile of daily air pollution value to the median value, whereas the extreme-low influence was estimated by comparing the 1st percentile of daily air pollution value to the median value. The elderly and adult were subgroups according to age (adult: 18-64 years; the elderly: $\geq$ 65 years).

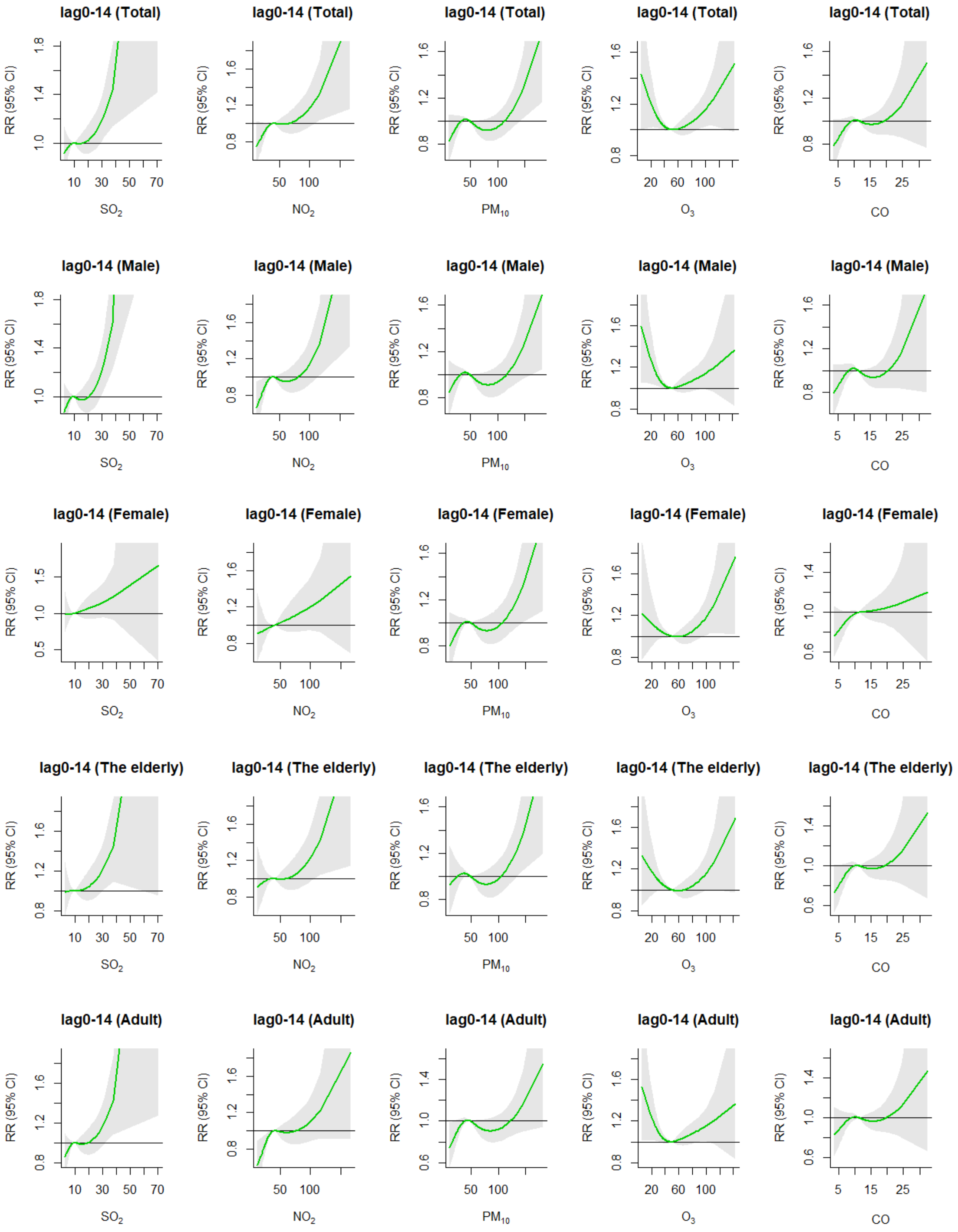

Figure S2 Summary of cumulative exposure-response curves on ischemic stroke for air pollution factors (SO_2, NO_2, PM_{10} and O_3) for all groups at lag0-14 using single-pollutant model in Shenzhen, 2008-2014. Male and female were subgroups according to gender. The elderly and adult were subgroups according to age (adult: 18-64 years; the elderly: ≥ 65 years).

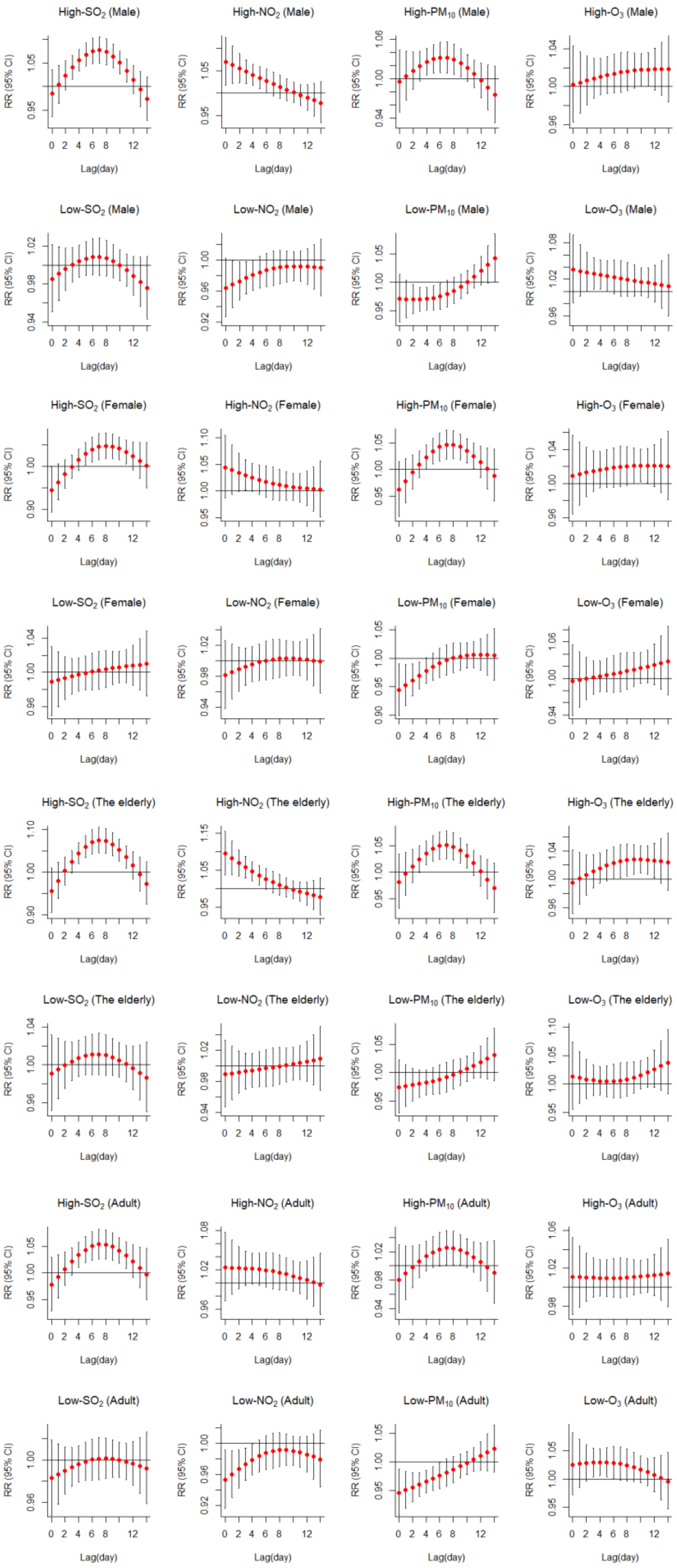
High-SO2 (Male)
High-NO2 (Male)
High-PM10 (Male)
High-O3 (Male)
Low-SO2 (Male)
Low-NO2 (Male)
Low-PM10 (Male)
Low-O3 (Male)
High-SO2 (Female)
High-NO2 (Female)
High-PM10 (Female)
High-O3 (Female)
Low-SO2 (Female)
Low-NO2 (Female)
Low-PM10 (Female)
Low-O3 (Female)
High-SO2 (The elderly)
High-NO2 (The elderly)
High-PM10 (The elderly)
High-O3 (The elderly)
Low-SO2 (The elderly)
Low-NO2 (The elderly)
Low-PM10 (The elderly)
Low-O3 (The elderly)
High-SO2 (Adult)
High-NO2 (Adult)
High-PM10 (Adult)
High-O3 (Adult)
Low-SO2 (Adult)
Low-NO2 (Adult)
Low-PM10 (Adult)
Low-O3 (Adult)
RR (95% CI)
Lag(day)

Figure S3 Summary of single day lag-response curves on ischemic stroke for air pollution factors (SO_2, NO_2, PM_{10} and O_3) for subgroups at different lags using two-pollutant model in Shenzhen, 2008-2014. The extreme-high influence was estimated by the *RR* of ischemic stroke by comparing the 99th percentile of daily air pollution value to the median value, whereas the extreme-low influence was estimated by comparing the 1st percentile of daily air pollution value to the median value. The elderly and adult were subgroups according to age (adult: 18-64 years; the elderly: ≥ 65 years).

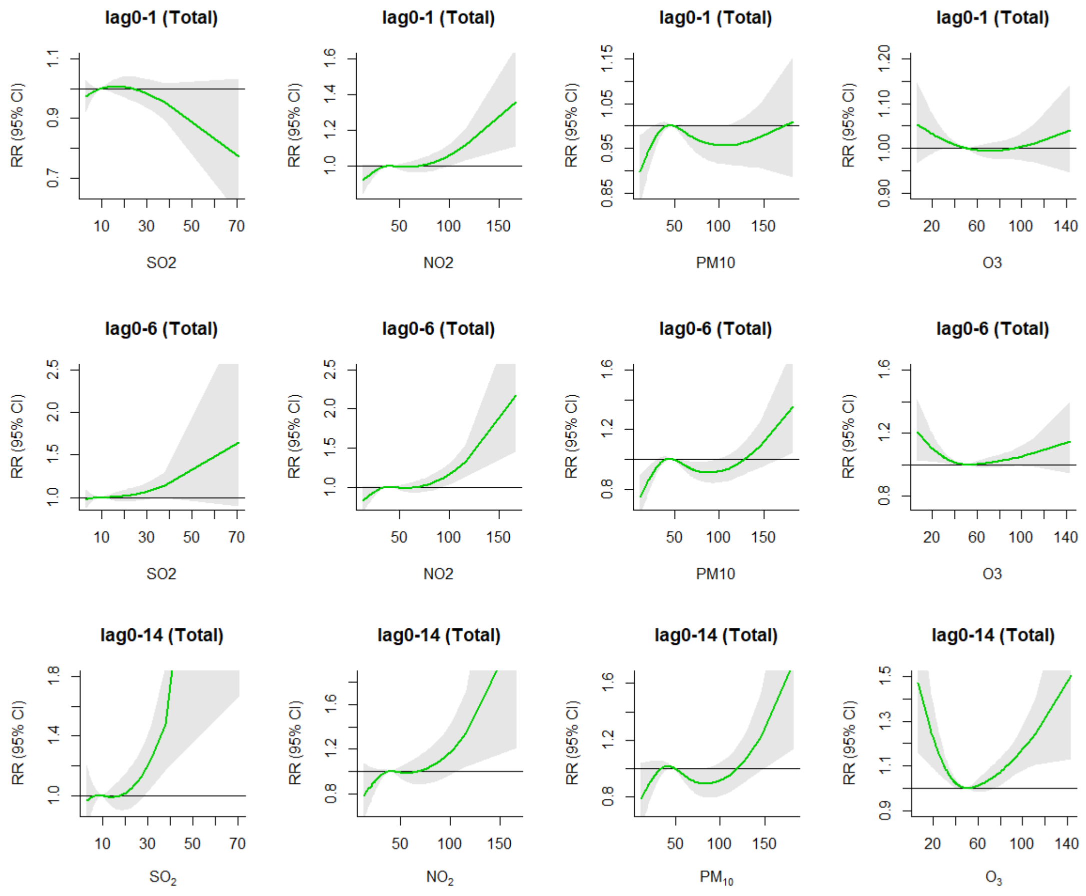

Figure S4 Summary of cumulative exposure-response curves on ischemic stroke for air pollution factors (SO_2, NO_2, PM_{10} and O_3) for total cases at lag0-1, lag0-6 and lag0-14 using two-pollutant model in Shenzhen, 2008-2014.

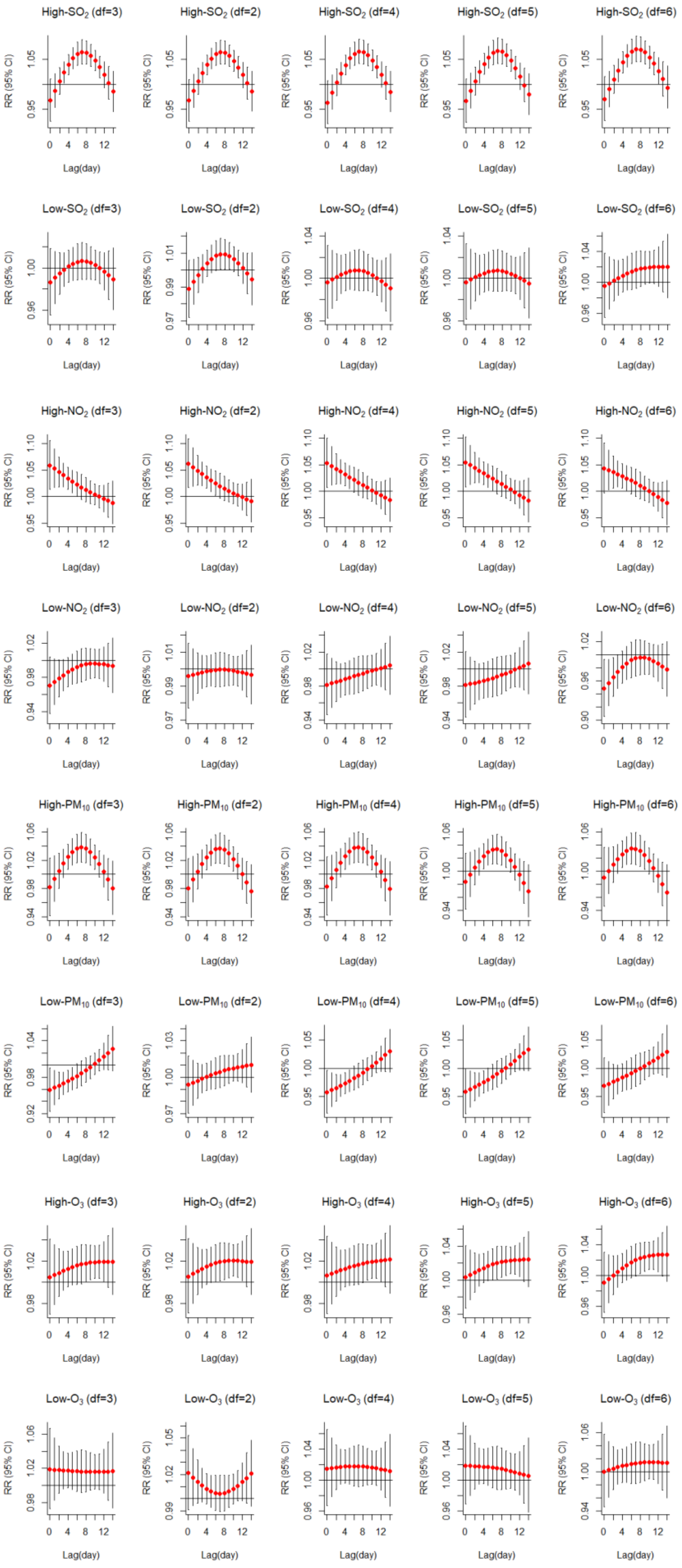
High-SO_2 (df=3)
High-SO_2 (df=2)
High-SO_2 (df=4)
High-SO_2 (df=5)
High-SO_2 (df=6)
Low-SO_2 (df=3)
Low-SO_2 (df=2)
Low-SO_2 (df=4)
Low-SO_2 (df=5)
Low-SO_2 (df=6)
High-NO_2 (df=3)
High-NO_2 (df=2)
High-NO_2 (df=4)
High-NO_2 (df=5)
High-NO_2 (df=6)
Low-NO_2 (df=3)
Low-NO_2 (df=2)
Low-NO_2 (df=4)
Low-NO_2 (df=5)
Low-NO_2 (df=6)
High-PM_{10} (df=3)
High-PM_{10} (df=2)
High-PM_{10} (df=4)
High-PM_{10} (df=5)
High-PM_{10} (df=6)
Low-PM_{10} (df=3)
Low-PM_{10} (df=2)
Low-PM_{10} (df=4)
Low-PM_{10} (df=5)
Low-PM_{10} (df=6)
High-O_3 (df=3)
High-O_3 (df=2)
High-O_3 (df=4)
High-O_3 (df=5)
High-O_3 (df=6)
Low-O_3 (df=3)
Low-O_3 (df=2)
Low-O_3 (df=4)
Low-O_3 (df=5)
Low-O_3 (df=6)
RR (95% CI)
Lag(day)
0 4 8 12

Figure S5 Summary of single day lag-response curves on ischemic stroke for air pollution factors (SO_2, NO_2, PM_{10} and O_3) for total cases at different lags using two-pollutant model by changing the df (2-6) of air pollution in Shenzhen, 2008-2014. The extreme-high influence was estimated by the *RR* of ischemic stroke by comparing the 99th percentile of daily air pollution value to the median value, whereas the extreme-low influence was estimated by comparing the 1st percentile of daily air pollution value to the median value.

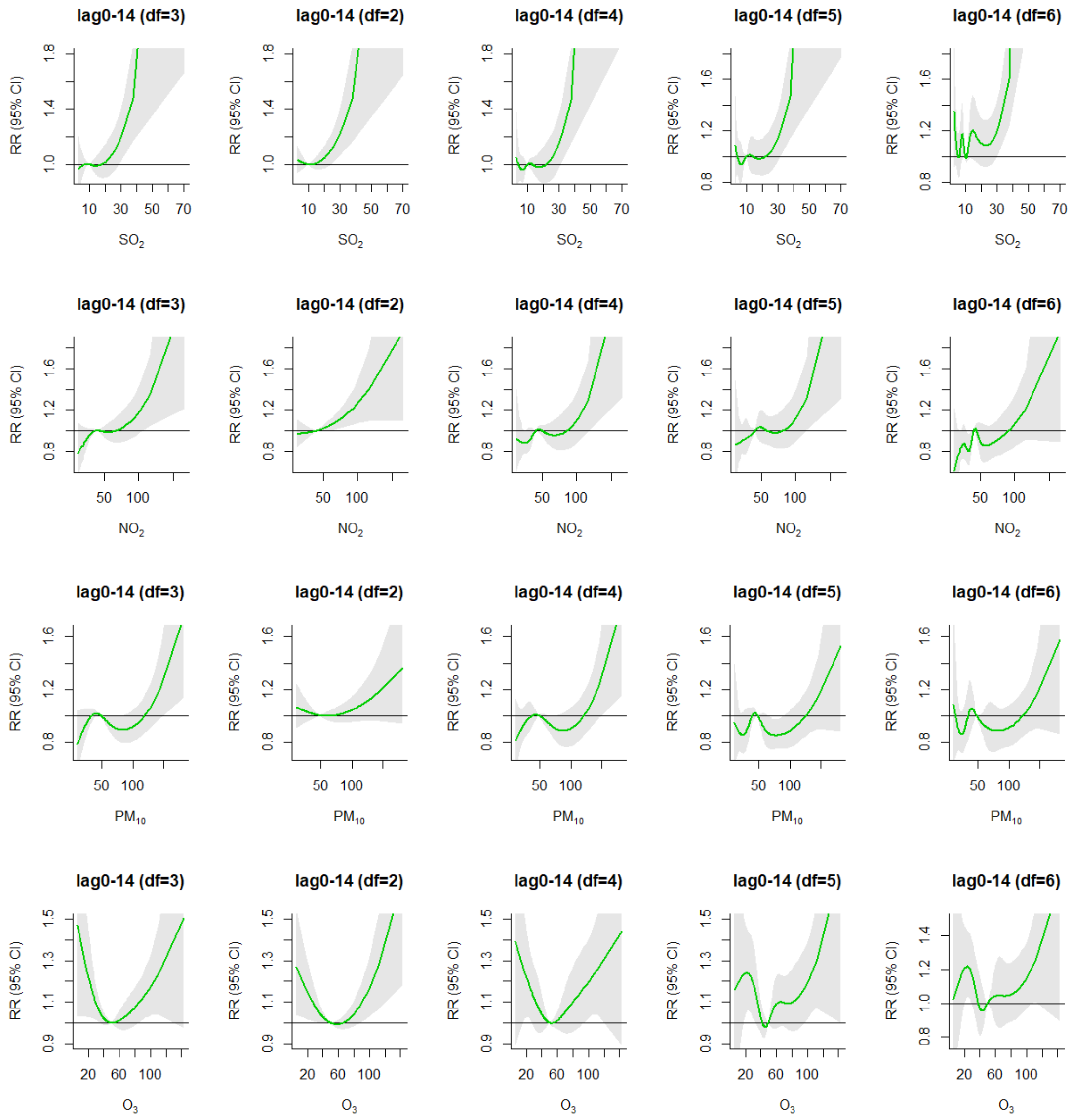

Figure S6 Summary of cumulative exposure-response curves on ischemic stroke for air pollution factors (SO_2, NO_2, PM_{10} and O_3) for total cases at lag0-14 using two-pollutant model by changing the df (2-6) of air pollution in Shenzhen, 2008-2014.

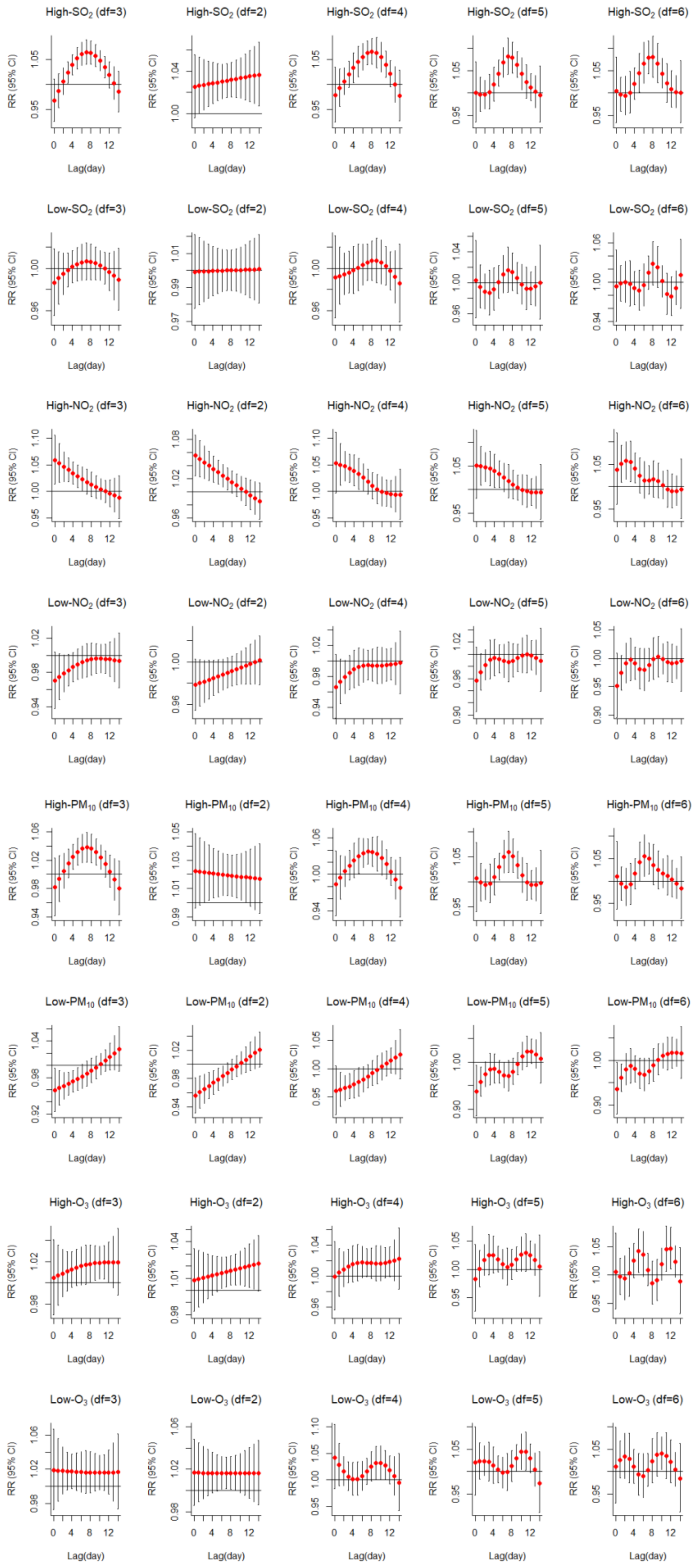
High-SO_2 (df=3)
High-SO_2 (df=2)
High-SO_2 (df=4)
High-SO_2 (df=5)
High-SO_2 (df=6)
Low-SO_2 (df=3)
Low-SO_2 (df=2)
Low-SO_2 (df=4)
Low-SO_2 (df=5)
Low-SO_2 (df=6)
High-NO_2 (df=3)
High-NO_2 (df=2)
High-NO_2 (df=4)
High-NO_2 (df=5)
High-NO_2 (df=6)
Low-NO_2 (df=3)
Low-NO_2 (df=2)
Low-NO_2 (df=4)
Low-NO_2 (df=5)
Low-NO_2 (df=6)
High-PM_{10} (df=3)
High-PM_{10} (df=2)
High-PM_{10} (df=4)
High-PM_{10} (df=5)
High-PM_{10} (df=6)
Low-PM_{10} (df=3)
Low-PM_{10} (df=2)
Low-PM_{10} (df=4)
Low-PM_{10} (df=5)
Low-PM_{10} (df=6)
High-O_3 (df=3)
High-O_3 (df=2)
High-O_3 (df=4)
High-O_3 (df=5)
High-O_3 (df=6)
Low-O_3 (df=3)
Low-O_3 (df=2)
Low-O_3 (df=4)
Low-O_3 (df=5)
Low-O_3 (df=6)
RR (95% CI)
Lag(day)

Figure S7 Summary of single day lag-response curves on ischemic stroke for air pollution factors (SO_2, NO_2, PM_{10} and O_3) for total cases at different lags using two-pollutant model by changing the df (2-6) of lag space in Shenzhen, 2008-2014. The extreme-high influence was estimated by the *RR* of ischemic stroke by comparing the 99th percentile of daily air pollution value to the median value, whereas the extreme-low influence was estimated by comparing the 1st percentile of daily air pollution value to the median value.

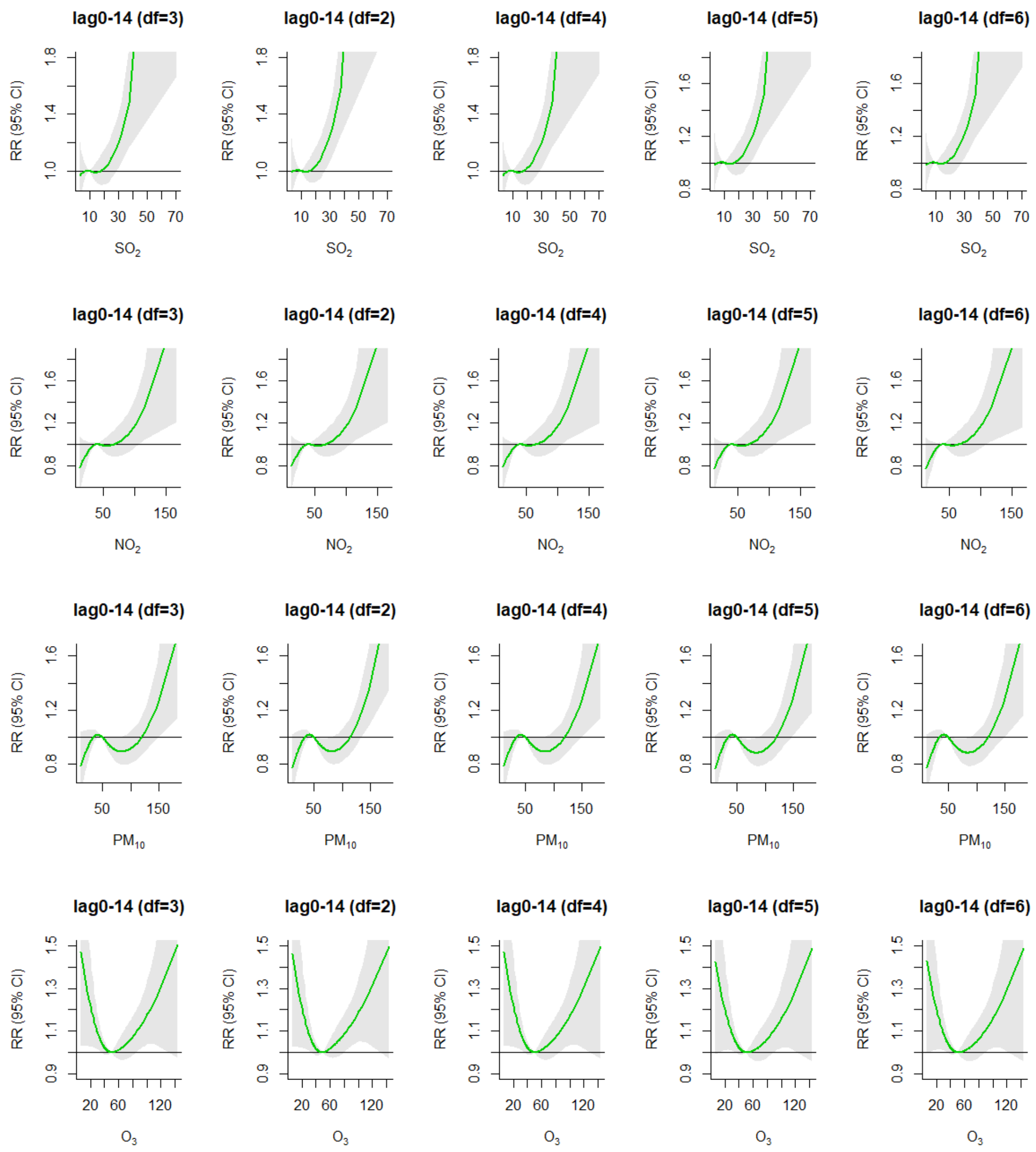

Figure S8 Summary of cumulative exposure-response curves on ischemic stroke for air pollution factors (SO_2, NO_2, PM_{10} and O_3) for total cases at lag0-14 using two-pollutant model by changing the df (2-6) of lag space in Shenzhen, 2008-2014.

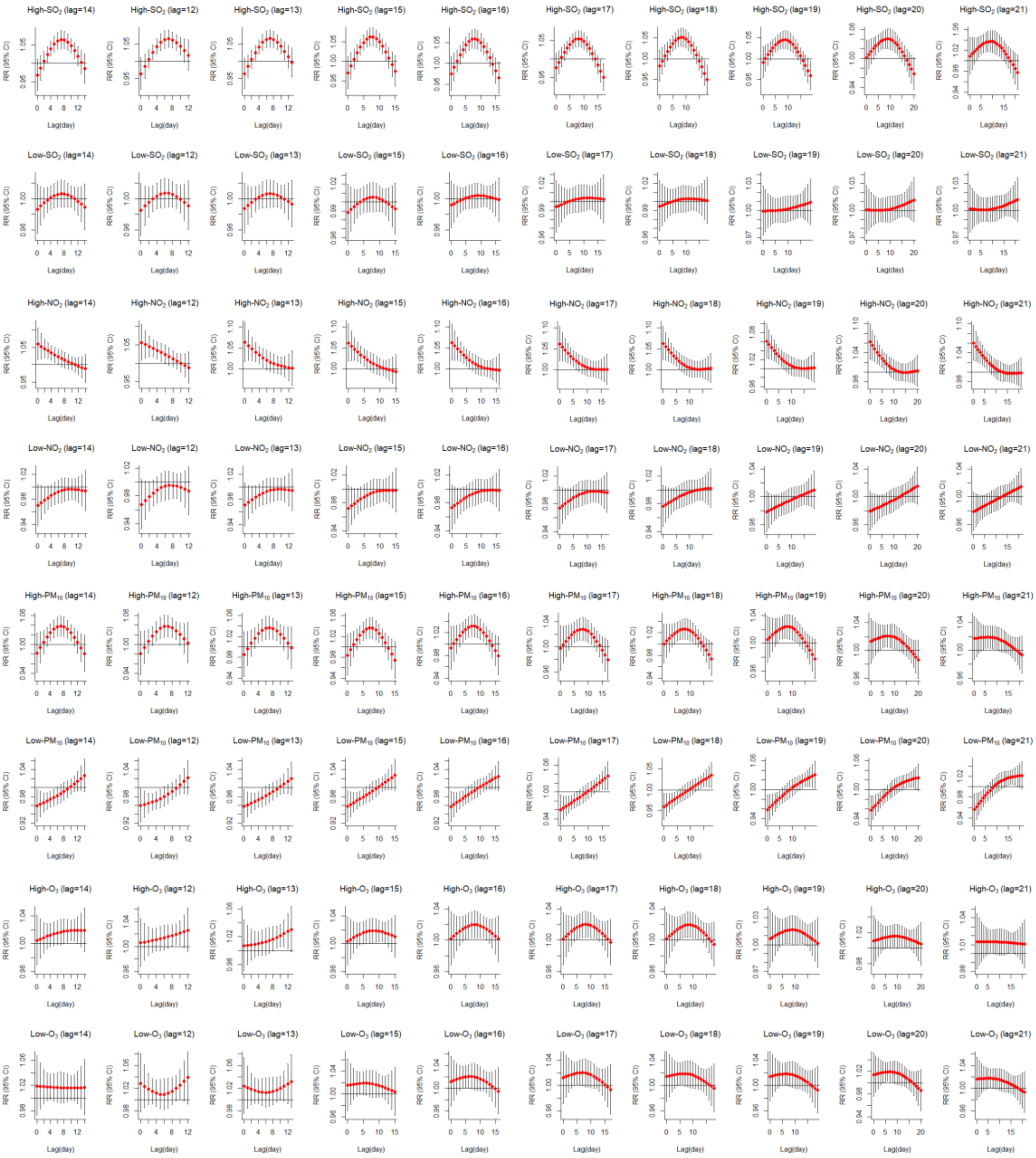

Figure S9 Summary of single day lag-response curves on ischemic stroke for air pollution factors (SO_2, NO_2, PM_{10} and O_3) for total cases at different lags using two-pollutant model by changing the maximum lag days (12-21 days) for air pollution in Shenzhen, 2008-2014. The extreme-high influence was estimated by the *RR* of ischemic stroke by comparing the 99th percentile of daily air pollution value to the median value, whereas the extreme-low influence was estimated by comparing the 1st percentile of daily air pollution value to the median value.

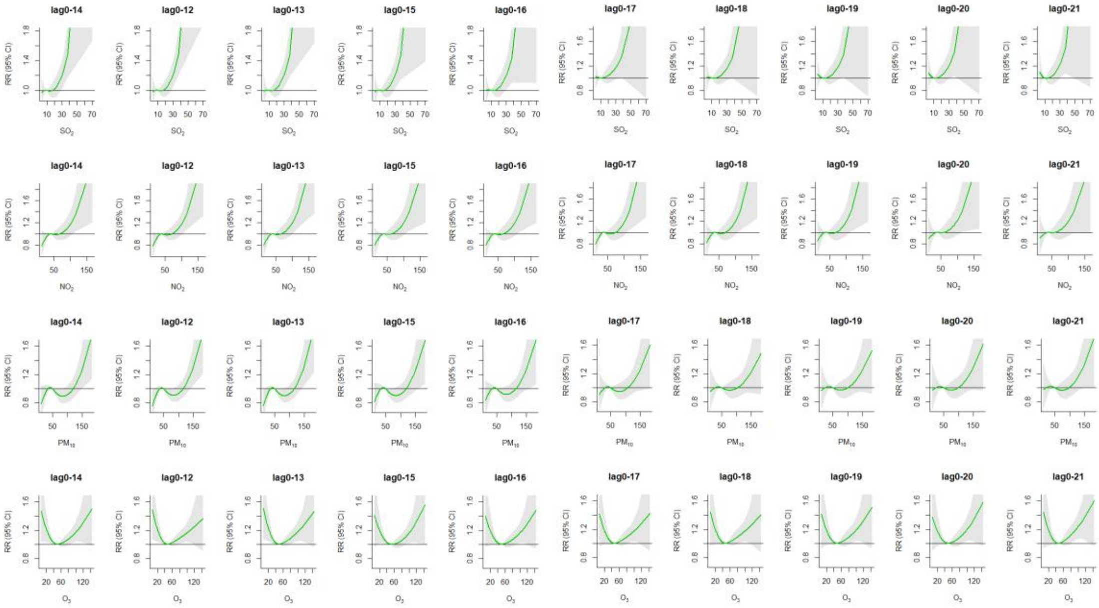

Figure S10 Summary of cumulative exposure-response curves on ischemic stroke for air pollution factors (SO_2, NO_2, PM_{10} and O_3) for total cases at lag0-14 using two-pollutant model by changing the maximum lag days (12-21 days) for air pollution in Shenzhen, 2008-2014.
